# Evolutionary evidence for multi-host transmission of cetacean morbillivirus

**DOI:** 10.1038/s41426-018-0207-x

**Published:** 2018-12-05

**Authors:** Wendy K. Jo, Jochen Kruppa, Andre Habierski, Marco van de Bildt, Sandro Mazzariol, Giovanni Di Guardo, Ursula Siebert, Thijs Kuiken, Klaus Jung, Albert Osterhaus, Martin Ludlow

**Affiliations:** 1 Research Center for Emerging Infections and Zoonoses, University of Veterinary Medicine Hannover, 30559 Hannover, Germany; 2 Institute for Animal Breeding and Genetics, University of Veterinary Medicine Hannover, 30559 Hannover, Germany; 3 Department of Viroscience, Erasmus Medical Center, 3015 CE Rotterdam, The Netherlands; 4 Department of Comparative Biomedicine and Food Science, University of Padova, 35020 Legnaro, Padua Italy; 5 Faculty of Veterinary Medicine, University of Teramo, 64100 Teramo, Italy; 6 Institute for Terrestrial and Aquatic Wildlife Research, University of Veterinary Medicine Hannover, 25761 Büsum, Germany

## Abstract

Cetacean morbillivirus (CeMV) has emerged as the pathogen that poses the greatest risk of triggering epizootics in cetacean populations worldwide, and has a high propensity for interspecies transmission, including sporadic infection of seals. In this study, we investigated the evolutionary history of CeMV by deep sequencing wild-type viruses from tissue samples representing cetacean species with different spatiotemporal origins. Bayesian phylogeographic analysis generated an estimated evolutionary rate of 2.34 × 10^−4^ nucleotide substitutions/site/year and showed that CeMV evolutionary dynamics are neither host-restricted nor location-restricted. Moreover, the dolphin morbillivirus strain of CeMV has undergone purifying selection without evidence of species-specific mutations. Cell-to-cell fusion and growth kinetics assays demonstrated that CeMV can use both dolphin and seal CD150 as a cellular receptor. Thus, it appears that CeMV can readily spread among multiple cetacean populations and may pose an additional spillover risk to seals.

## Introduction

Cetacean morbillivirus (CeMV) is known to infect a wide range of cetacean species from both Odontoceti (toothed whales) and Mysticeti (baleen whales) subgroups^1^, including threatened and endangered species, such as the fin whale (*Balaenoptera physalus*) and sperm whale (*Physeter macrocephalus*). This emerging morbillivirus causes variable degrees of morbidity and mortality and thus may have a major impact on the demography and conservation of cetacean populations. Five distinct strains of CeMV have now been documented^2–6^ since the identification of porpoise morbillivirus (PMV) in stranded harbor porpoises (*Phocoena phocoena*) in Ireland and the Netherlands in 1988 and 1990, respectively^2,7^. Dolphin morbillivirus (DMV) is the best characterized strain, having caused several epizootics in addition to isolated cases that were retrospectively identified using molecular and pathological diagnostic protocols. The first documented CeMV outbreak killed thousands of striped dolphins (*Stenella coeruleoalba*) in the Mediterranean Sea from 1990 to 1992^3^. However, CeMV was later identified as the etiological agent responsible for an earlier epizootic in bottlenose dolphins (*Tursiops truncatus*) along the northwestern Atlantic coast in 1987–1988^8^. The trans-oceanic occurrence of CeMV infections continued with later outbreaks in the Gulf of Mexico in 1993–1994^9^, the Mediterranean Sea in 2006–2008^10^, the coast of South Australia in 2013^11^, along the eastern USA seaboard in 2013–2015^12^, and in southeastern Brazil in 2017^13^, causing mass die-offs of striped dolphins, bottlenose dolphins, Indo-Pacific bottlenose dolphins (*T. aduncus*), short-beaked common dolphins (*Delphinus delphis*), long-finned pilot whales (*Globicephala melas*), and Guiana dolphins (*Sotalia guianensis*). Additionally, DMV infections have been diagnosed in cases of fin whale and sperm whale strandings, possibly playing a role in their demise^14–16^. Pilot whale morbillivirus (PWMV) has been sporadically reported to have infected pilot whales (*G. melas* and *G. macrorhynchus*) in the northeastern and northwestern Atlantic^4,17^. In recent years, two novel CeMV strains have been identified, namely beaked whale morbillivirus (BWMV) in the central Pacific^5^ and a more divergent strain, which is called Guiana dolphin morbillivirus (GDMV) in this study for simplification purposes, that was detected in Guiana and Indo-Pacific bottlenose dolphins found stranded along the Brazilian^6^ and West Australian coasts^18^, respectively.

CeMV is an enveloped negative-sense single-stranded RNA virus in the genus Morbillivirus within the family *Paramyxoviridae* along with measles virus (MV, the prototypic species), the recently eradicated rinderpest virus (RPV), peste des petits ruminants virus (PPRV), canine distemper virus (CDV), phocine distemper virus (PDV), and feline morbillivirus (FeMV). A characteristic of animal morbilliviruses (i.e., RPV, PPRV, CDV, and CeMV) is a propensity for interspecies transmission. For instance, RPV infected multiple wild and domestic species of ungulates, whereas CDV can infect multiple carnivores and spread from carnivores to non-human primates, rodents, and artiodactyls^19^. In addition to multiple dolphin and whale species^1^, CeMV has also crossed the species barrier to infect marine mammals belonging to a different order. In 1997, DMV was associated with an epizootic that halved the already endangered population of Mediterranean monk seals (*Monachus monachus*) in Mauritania^20^, although it was later proposed that algal intoxication directly caused these deaths^21^. Consequently, the direct cause of this epizootic remains a matter of debate. Additional documented cases of CeMV infection of seal species include a monk seal in Greece^22^ and a captive harbor seal (*Phoca vitulina*) in Italy^23^ that were reported to be PMV-infected and DMV-infected, respectively. However, the host range of CeMV and potential barriers to cross-species infections remain to be further determined.

In common with other morbilliviruses, CeMV displays a tropism for lymphoid and epithelial cells, and induces typical lesions including bronchointerstitial pneumonia and lymphocytic depletion in lymphoid organs^3,6,14,16,17,24^. In addition, CeMV is neurotropic as non-suppurative encephalitis is commonly observed in infected cetaceans. Cell entry and spread of morbilliviruses is mediated by the viral fusion (F) and hemagglutinin (H) glycoproteins. Signaling lymphocyte activation molecule (SLAM/CD150) and poliovirus receptor-like 4 (PVRL-4/Nectin-4) have been previously identified as universal morbillivirus cellular receptors^25,26^. SLAM has been found in immune cells and appears to be the primary receptor for virus entry and spread to other tissues, whereas Nectin-4 is the primary receptor of epithelial cells and is important for virus exit and transmission. The route of transmission is usually through aerosol droplets as well as direct or indirect contact. In the case of cetaceans, virus dissemination in the population is speculated to occur via direct contact or aerogenic means^1^. It is assumed that morbillivirus excretion and transmission occur predominantly from virus-infected epithelial cells at mucosal surfaces. High levels of CeMV-positive epithelial cells have previously been detected in the respiratory and urogenital tracts of infected dolphins and whales^9,11,17^. Vertical transmission has also been discussed as another possible route of virus infection^14^.

Although a large number of case studies investigating CeMV epidemiology via serology and partial sequences have been reported^1^, no studies on CeMV evolution in relation to trans-oceanic viral spread based on whole genome sequences are available. In the present study, we have deep-sequenced European CeMV strains from a collection of historical and recent CeMV-infected tissue samples and virus isolates to investigate the evolutionary history of CeMV and to identify possible molecular signatures of host adaptation arising from interspecies virus transmission.

## Results

### Genome analyses of wild-type and laboratory-adapted CeMVs

**Fig. 1:**
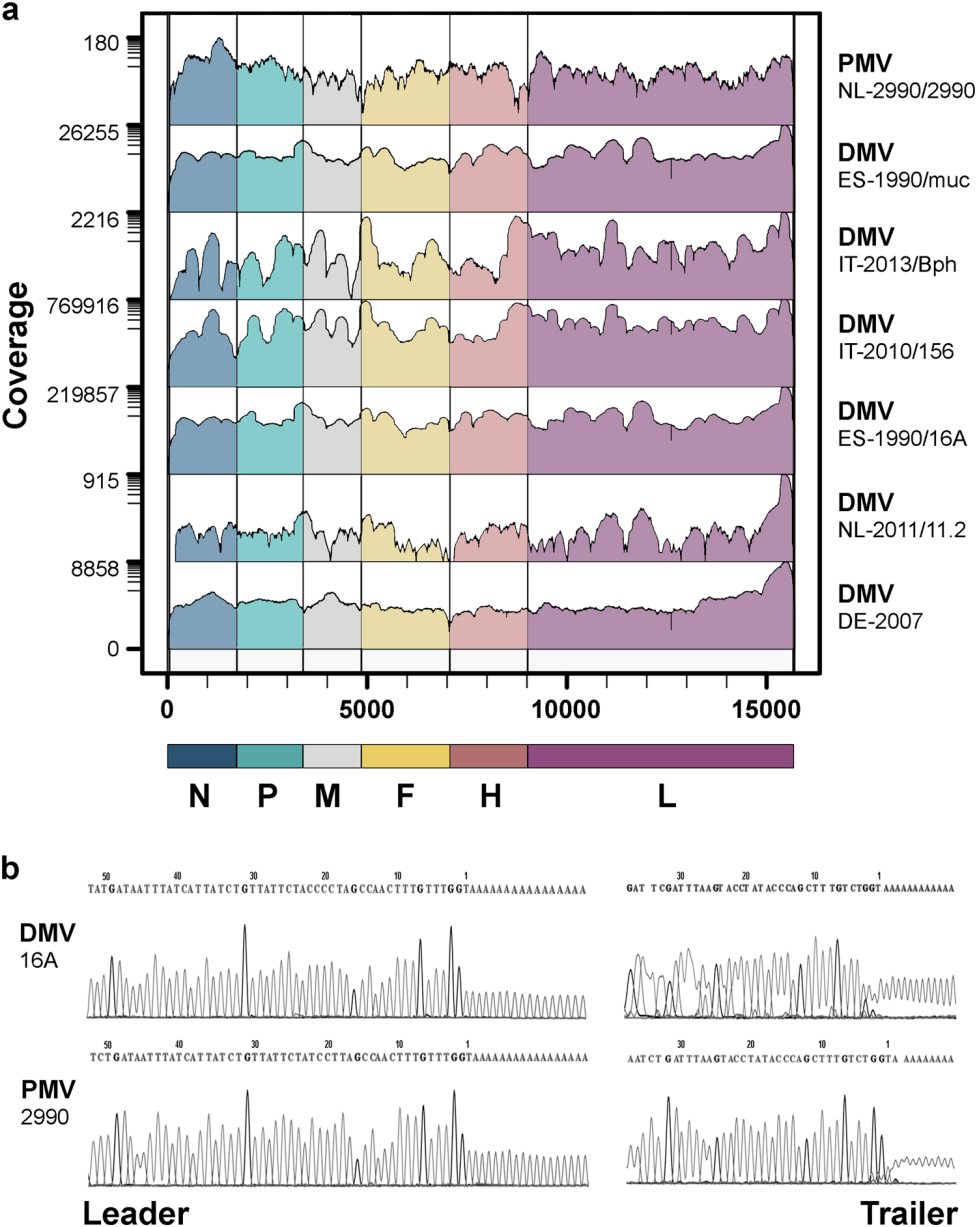
Recovery of full-length CeMV sequences by NGS and RACE. **a** Genome coverage of wild-type CeMVs. The scale on the left indicates sequencing depth and genome regions are color-coded according to CeMV gene positions. **b** Sequencing chromatograms of leader and trailer regions of wild-type CeMV strains DMV-16A and PMV-2990 using RACE

A collection of historical and recent CeMV-infected tissue samples from Europe was investigated along with cell culture-passaged CeMV strains (Table 1). Samples were first screened by real-time PCR (RT-PCR) to assess the viral load with Ct values ranging from 12 to 32. Complete CeMV genome sequences from tissue samples and virus isolates were generated by a combination of next generation sequencing (NGS) (Fig. 1a) and rapid amplification of cDNA ends (RACE; Fig. 1b and Supplementary Fig. S1). In addition, gap sequencing was performed for CeMV strains DMV-Bph, DMV-NL/LA/11.1, and PMV-53 due to low coverage in a number of regions. Host reference alignments of evaluated samples permitted confirmation of the identity of the different host species (dolphin or whale). An analysis of genome termini showed the presence of two nt changes at positions 5 (C → U) and 12 (G → A) of the leader sequence in all CeMV sequences compared to the lab-adapted 1990 DMV strain (GenBank Accession No. AJ608288) that was used as a reference genome in this study.

**Table 1:** Cetacean morbillivirus detection in cetacean samples from the North Sea and the Mediterranean Sea

CeMV strain	Variant	Stranding year	Location	Host	Sample material	RT-PCR (Ct)	NGS coverage	GenBank accession no.	References
PMV	Ulster/88	1988	Northern Ireland, U.K.	Harbor porpoise	Vero cells	NI	5149×	MH430942	2
DMV	16A	1990	Spain	Striped dolphin	Lung	12	6023×	MH430934	28
DMV	16A-cc	1990	Spain	Striped dolphin	Vero cells	13	27×	MH430932	This study
DMV	16A-cc-vdol	1990	Spain	Striped dolphin	Vero-dolSLAM cells	13	1188×	MH430933	This study
DMV	muc	1990	Spain	Striped dolphin	Lung	13	1248×	MH430935	28
DMV	muc-cc	1990	Spain	Striped dolphin	Vero cells	13	20×	MH430936	This study
PMV	53	1990	Netherlands	Harbor porpoise	Vero cells	NI	9120×	MH430943	7
PMV	2990	1990	Netherlands	Harbor porpoise	Brain	32	26×	MH430945	7
PMV	2990-cc	1990	Netherlands	Harbor porpoise	Vero cells	NI	11,933×	MH430944	This study
DMV	DE/2007	2007	Germany	White-beaked dolphin	Brain	19	283×	MH430940	48
DMV	156	2010	Italy	Striped dolphin	Lung	19	50,169×	MH430937	This study
DMV	DMV/LA/NL/11.2	2011	Netherlands	White-beaked dolphin	Lung	23	21×	MH430941	24
DMV	DMV_Bph	2013	Italy	Fin whale	Brain	32	144×	MH430938	14, 49
DMV	DK/16^a^	2016	Denmark	Fin whale	Lung	27	NI	MH430939	16

Ct value ≤ 40 = positive; Ct value > 40 = negative

*RT-PCR* reverse transcriptase PCR, *Ct value* threshold cycle, *NGS* next-generation sequencing, *NI* not investigated

^a^Genome obtained by Sanger sequencing

**Fig. 2:**
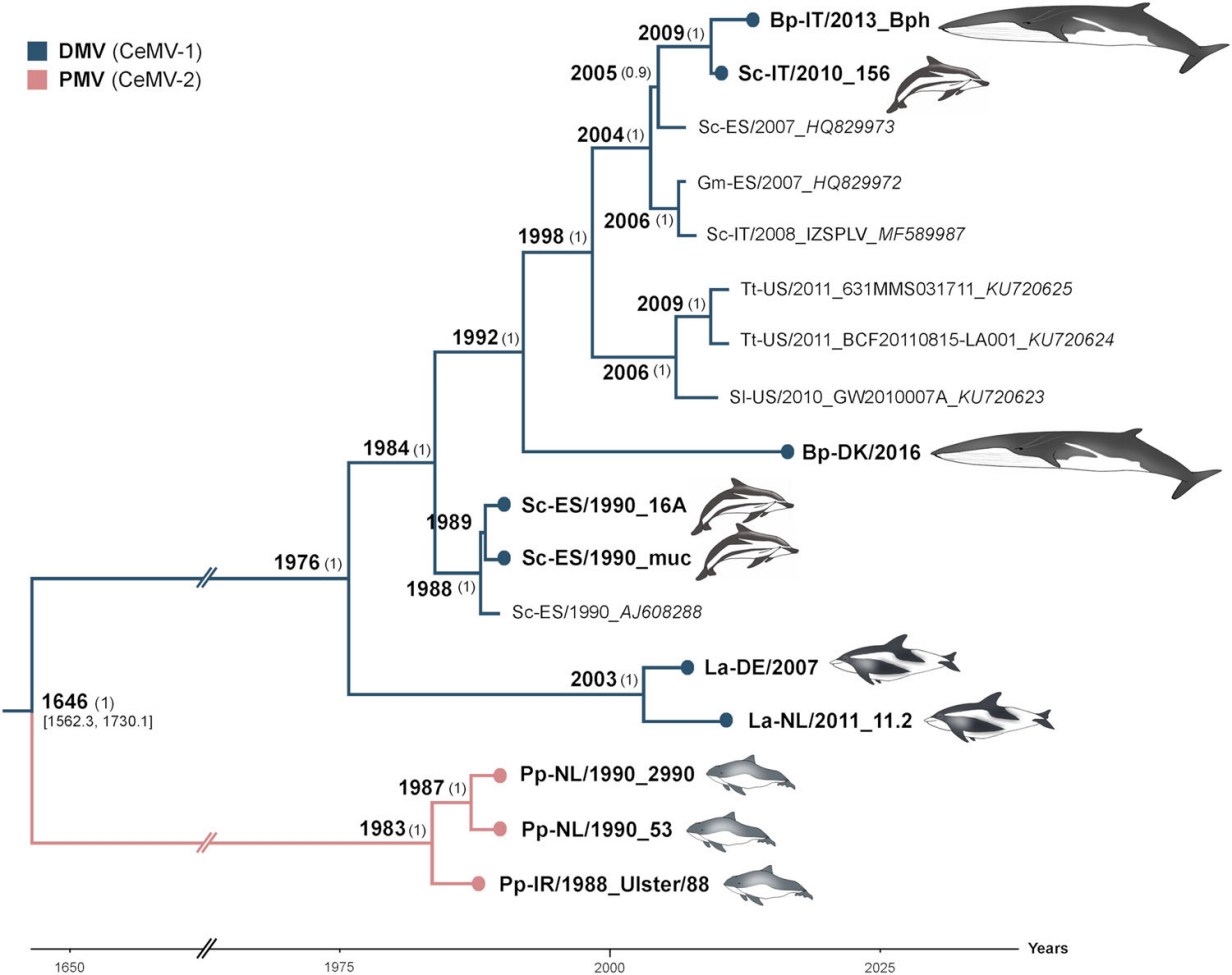
**Bayesian phylogenetic analysis of full-length CeMVs excluding L sequences.** Most common recent ancestor ages are presented at the nodes with posterior values > 0.7 shown in parenthesis. New CeMV genomes generated during this study are presented with circles at the tips. Branches were truncated for graphical reasons. The taxon names are presented as host-country/year of collection_variant_*GenBank accession number*. Branch colors represent virus strains. New CeMV variants and GenBank accession numbers Bph (MH430938), 156 (MH430937), DK/16 (MH430939), 16A (MH430934), muc (MH430935), DE/2007 (MH430940), NL/11.2 (MH430941), 2990 (MH430945), 53 (MH430943), and Ulster/88 (MH430942). Bp *Balaenoptera physalus*, La *Lagenorhynchus albirostris*, Pp *Phocoena phocoena*, Sc *Stenella coeruleoalba*, Sl *Stenella longirostris*, and Tt *Tursiops truncatus*

The genetic variation within individual CeMV lineages was low, with a pairwise identity distance of 98.3–99.9% between DMV strains and 99–7-99.8% between PMV strains. In contrast, an 86.3–86.8% pairwise identity distance was observed between DMV and PMV strains. A Bayesian estimation of the substitution rate of CeMV was 2.34 × 10^−4^ nucleotide substitutions/site/year (subs/site/year) with 95% of the highest posterior density intervals at 1.86–2.83 × 10^−^^4^ subs/site/year. The substitution rates of individual coding sequences for N, P, M, F, H, and L resulted in a similar range (Supplementary Fig. S2). However, the non-coding regions were estimated to have a higher mutation rate of 5.58 × 10^−4^ subs/site/year, as these regions are usually less often subjected to selective pressures in comparison to coding regions. We excluded L gene sequences from subsequent Bayesian analyses as these were absent from the partial genome sequences of two published DMVs from the 2006–2008 outbreak^27^. Moreover, the L gene sequence of the reference variant was excluded from the analyses due to the presence of many unique mutations, which may be artefactual, in comparison to all other DMV strains, including two new L gene sequences derived from dolphin tissues obtained from the same epizootic in 1990 (GenBank Accession Nos. MH430934-35). Non-synonymous substitutions in this variant accounted for 41% of the total amino acid (aa) changes in L (Supplementary Fig. S3). The tree topology was not changed when these sequences were taken out for analyses of complete DMV genomes (Supplementary Fig. S4). Phylogenetic analyses based on Bayesian inference suggested that DMV and PMV shared a most recent common ancestor (MRCA) approximately 400 years ago (Fig. 2). Moreover, the DMV strain responsible for the mass die-off in the Mediterranean Sea in 1990–1992 had a more basal position relative to other DMV variants, which emerged in subsequent years in the Mediterranean Sea (isolate_GenBank Accession Nos. HQ829972, HQ829973, IZSPLV_MF589987, 156_MH430937, Bph_MH430938), North Sea (isolate_GenBank Accession No. DK/16_MH430939), and the Gulf of Mexico (GenBank accession nos. KU720623-25). A full-length DMV sequence recovered from a fin whale stranded on the Danish coast in 2016 was also located within this DMV clade. However, additional North Sea variants from white-beaked dolphins (GenBank accession nos. DE/2007_MH430940 and NL/11.2_MH430941) were exceptions, as they shared a common ancestor with this Mediterranean DMV variant in 1976 (Fig. 2).

**Fig. 3:**
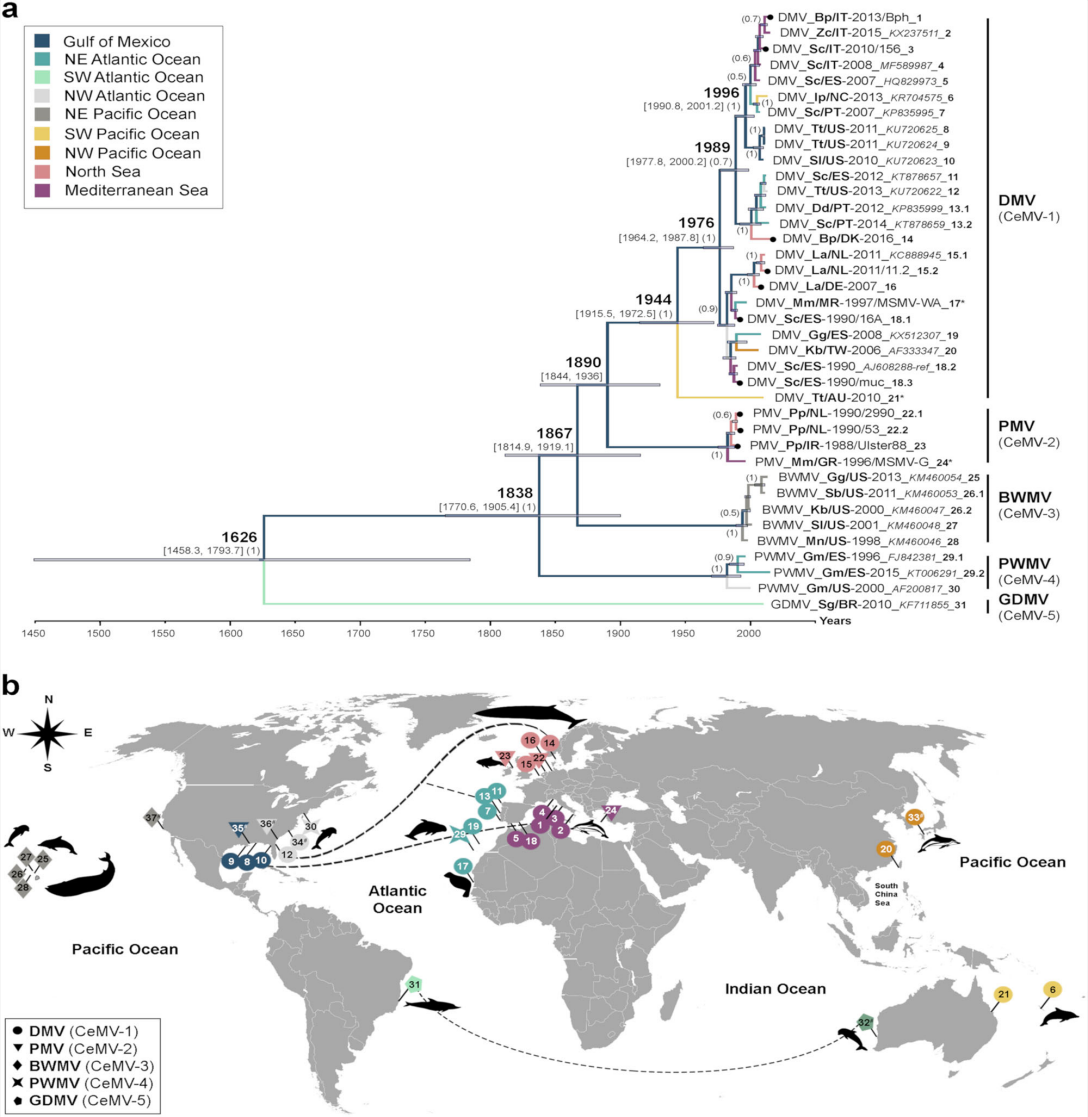
Phylogeography of CeMV. **a** Bayesian phylogenetic analysis of partial P genes (400 bp). Most common recent ancestor ages are presented at the nodes in cursive with 95% highest posterior density interval values in brackets and as gray horizontal bars. Posterior values > 0.5 are shown in parenthesis. CeMV genomes in this study are presented with black circles at the tips. Each branch is color-coded according to the ocean/sea in which the cetaceans were found. The taxon names are presented as virus_host/Country-year of collection/variant_*GenBank accession number*_ID for **b**. Sequences 17* and 24* were extracted from a published paper^22^. Sequence 21* was kindly provided by Dr. Stone and Jianning Wang. Host abbreviations: Bp *Balaenoptera physalus*, Dd *Delphinus delphis*, Gg *Grampus griseus*, Gm *Globicephala melas*, Ip *Indopacetus pacificus*, Kb *Kogia breviceps*, La *Lagenorhynchus albirostris*, Mm *Monachus monachus*, Mn *Megaptera novaeangliae*, Pp *Phocoena phocoena*, Sb *Steno bredanensis*, Sc *Stenella coeruleoalba*, Sg *Sotalia guianensis*, Sl *Stenella longirostris*, Tt *Tursiops truncatus*, and Zc *Ziphius cavirostris*. **b** A world map of CeMV migration using sequences from **a**. Locations of viruses in the map are relative and were obtained from the publications in which the sequences were published. The hashtag (#) indicates no sequence availability (No. 32: CeMV-5 from AU/2010 and No. 33: CeMV-1_JP/1999). Proposed virus migratory routes (dashed lines) were based on sequence clades from the phylogenetic tree in **a**

Phylogenetic analysis of individual genes displayed different tree topologies (Supplementary Fig. S5), of which the F and H gene phylogenies best resembled the tree derived from full-length CeMV sequences. Not surprisingly, this analysis is indicative of differing selective pressures in shaping the evolution of CeMV, in which the F and H genes apparently have key roles. Nevertheless, a distinction between different strains can still be made using more conserved viral genes. Therefore, we extended our analysis of global CeMV distribution patterns by examining a much larger data set of available partial CeMV P gene sequences. Phylogeographic analysis of 38 partial P gene sequences, which were previously deposited in GenBank or derived from this study, demonstrated that CeMV strains are neither host-specific nor location-specific (Fig. 3a), as also suggested by the full-length sequence analyses. The multi-host transmission of CeMV among cetacean species is best represented by DMV and BWMV, which have been identified in multiple odontocete and mysticete species^3–5,14,15^. The trans-oceanic spread of DMV is also readily apparent in closely related strains detected in the Gulf of Mexico, the Mediterranean Sea (Figs. 2 and 3, sequences 8–10 and 1–5), the east and west coasts of the North Atlantic Ocean and the North Sea (Fig. 3, sequences 11–14). Different virus dispersal routes through the Atlantic Ocean and from the Atlantic to the Indian Oceans are proposed (Fig. 3b). Furthermore, each CeMV strain formed a distinctive clade grouping the sequences of DMV (CeMV-1), PMV (CeMV-2), BWMV (CeMV-3), PWMV (CeMV-4), and GDMV (CeMV-5; Fig. 3a). This last strain was the most basal and divergent, sharing an MRCA with other CeMV strains a few hundred years ago. The MRCA shared by DMV and PMV was found to have different dates upon comparison of either complete or partial P gene sequences (Figs. 2 and 3a). While full-length genome sequence analysis would be expected to be more accurate, only two strains (DMV and PMV) could be included. In contrast, analysis of partial P gene sequences included sequences from all five known CeMV strains. However, given the differences in the evolutionary rate of the P gene compared with full-genome sequences, an analysis based on partial sequence may be less representative.

### Identification of molecular signatures of CeMV adaptation associated with in vitro replication

Given that historical morbillivirus sequences, including CeMV, were generated using virus isolates, we have also examined the effects of virus isolation and passaging on the resulting consensus genome sequences. Complete genome sequences from DMV-16A and DMV-muc, which were first isolated and passaged in Vero cells 25 years ago^28^, were compared to consensus sequences of the same viruses generated directly from original stored tissue samples. In addition, we generated Vero cells expressing SLAM derived from a Pacific white-sided dolphin (*Lagenorhynchus obliquidens*; Vero-dolSLAM) to investigate if expression of a natural CeMV cellular receptor better maintains the authentic consensus virus sequence. These cells were used to re-isolate DMV-16A from a stored tissue sample collected during the CeMV epizootic in 1990. A single large syncytium was observed 2–3 days following infection, and the virus was subsequently passaged four times before NGS was used to generate a complete consensus viral sequence.

**Fig. 4:**
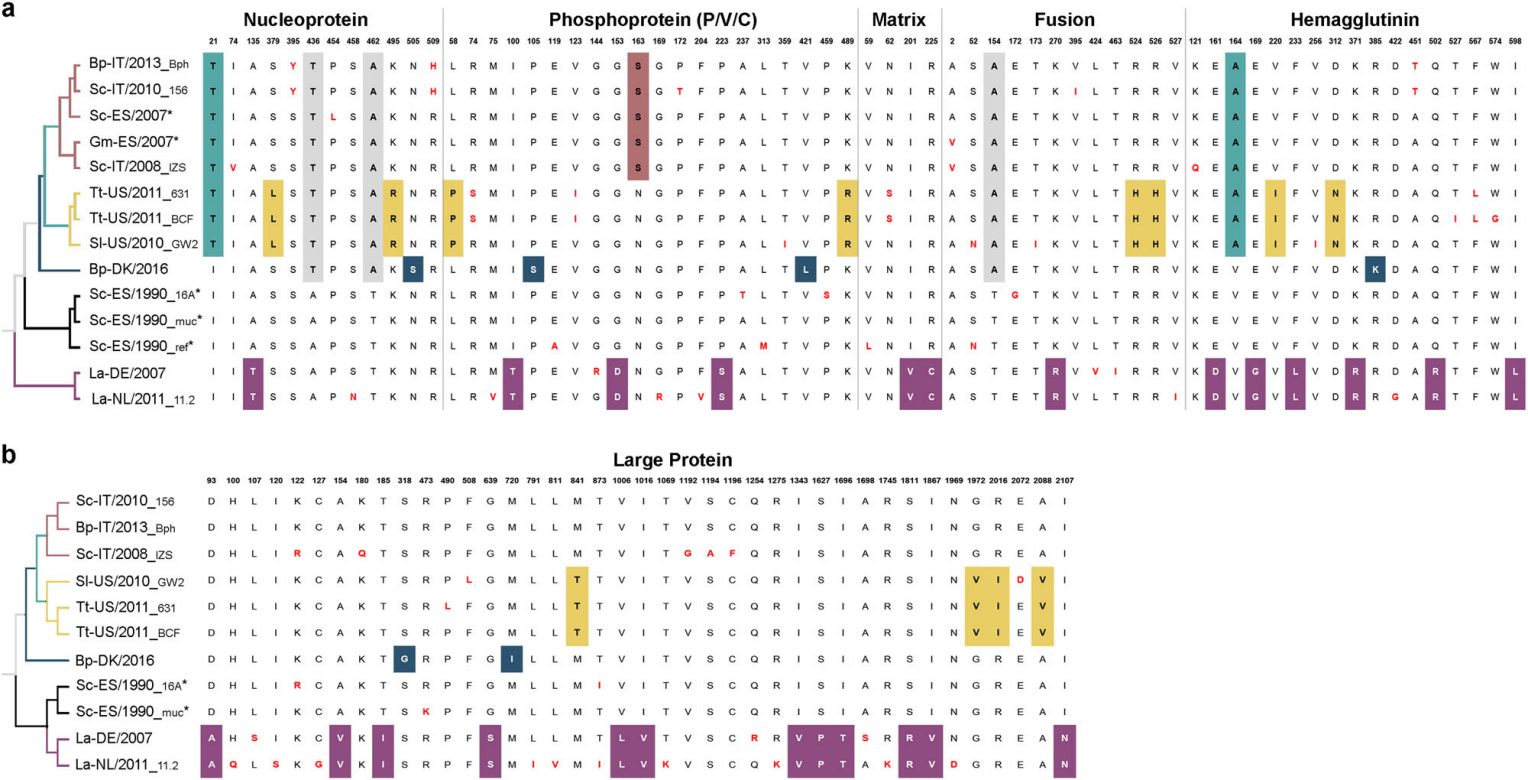
**Amino acid changes between DMV variants.** Defined molecular signatures of each monophyletic group are color-colored. Single changes not representing the group are shown in red. The asterisk denotes sequences obtained from outbreaks. The taxon names are presented as host-Country/year of collection_variant (first three characters). **a** comparison of amino acid changes in the nucleoprotein, phosphoprotein, matrix, fusion and hemagglutinin in the complete set of DMV genomes. **b** Comparison of amino acid changes in the L protein. DMV variants and GenBank accession numbers: Bph (MH430938), 156 (MH430937), Sc-ES/2007 (HQ829973), Gm-ES/2007(HQ829972) IZSPLV (MF589987), 631MMS031711 (KU720625); BCF20110815-LA001 (KU720624), GW2010007A (KU720623), DK/2016 (MH430939), 16A (MH430934), muc (MH430935), ref (AJ608288), DE/2007 (MH430940), and 11.2 (MH430941). Host abbreviations: Bp *Balaenoptera physalus*, La *Lagenorhynchus albirostris*, Pp *Phocoena phocoena*, Sc *Stenella coeruleoalba*, Sl *Stenella longirostris*, and Tt *Tursiops truncatus*

Sequence analysis of wild-type viruses present in tissue samples and viruses passaged in Vero cells and/or Vero-dolSLAM cells revealed minimal genetic variation (Table 2). The isolate DMV-16A grown in Vero-dolSLAM cells showed less variation from the wild-type consensus sequence than the same variant grown in Vero cells. Mutations S459P in P and I873T in L emerged in both isolates following in vitro passage. However, due to the presence of these substitutions in other wild-type DMV sequences (Fig. 4), we used the raw NGS data to calculate the frequencies of these variants in the virus population. Whereas the Vero cell isolates homogeneously expressed 459P in the P gene and 873T in the L gene, these positions were heterogeneous in the wild-type tissue-derived DMV-16A sequence (P-459: S = 53%, P = 46%; L-873: I = 52%, T = 47%), which was less heterogeneous in the DMV-16A isolated from Vero-dolSLAM (P-459: S = 1%, P = 99%; L-873: I = 4%, T = 96%) and showing different degrees of heterogeneity in all other wild-type sequences for S/P at 459 (DMV-muc: S = 41%, P = 48%; DMV-156: S = 18%, T = 81%; DMV-Bph: S = 3% P = 97%; DMV-DE/2007: S = 1%, P = 98%; and DMV/LA/NL/11.2: S = 40%, P = 60%) in the P gene and isoleucine and threonine at 873 (DMV-muc: I = 49%, T = 51%; DMV-156: I = 29%, T = 71%; DMV-Bph: I = 27%, T = 73%; DMV-DE/2007: I = 2%, T = 95%; and DMV/LA/NL/11.2: I = 65%, T = 35%) in the L gene.

**Table 2:** Sequence variation between wild-type and isolated CeMV strains

Strain-variant	Gene	Position (ORF)	Codon	Wild-type	Vero	V. dolSLAM	Mutation
DMV-16A	Leader	26	—	a	a/t	a	g.26A>T
	P	693	231	c	g	c	*dS*
	P	1375	459	Ser^1^	Pro	Pro	S459P
	M	310	104	Thr	Ala	Thr	T104A
	M	502	168	Asn	His	Asn	N168H
	F	1339	447	Arg	Gly	Arg	R447G
	L	2618	873	Ile^2^	Thr	Thr	I873T
DMV-muc	Leader	11	—	g	a	—	g.11G>A
	M	523	175	Arg	Gly	—	R175G
	M	681	227	Lys	Asn	—	K227N
PMV-2990	M	265	89	Glu	Lys	—	E89K
	H	224	75	Asn	Asn	—	S75N
	L	406	126	Trp	Trp	—	G126W
	L	4125	1375	t	c	—	*dS*

*dS* Synonymous substitution

Upon comparison of whole genome sequences of in vitro isolates of DMV-16A, DMV-muc, and PMV-2990 with analogous sequences generated from original tissue samples, we did not detect common mutations that had arisen as a result of cell culture adaptation. Instead, we identified changes in the individual variants that have been previously detected in MV studies^29–31^. These changes include the nucleotide (nt) substitution g.26A>T^29^ and aa substitutions in the M protein, namely E89K (PMV-2990)^31^ and R175G (DMV-muc)^30^. An analysis of the genome sequence of DMV-16A, which had been passaged in Vero cells, showed the presence of T at nt 26, although A was still present as a minor variant. Upon the inclusion of other morbilliviruses in an alignment of genome termini, wild-types had an A at nt 26, whereas vaccine strains had a T at this position (Supplementary Fig. S1).

### The capacity of CeMV to cross-species barriers

Alignment of full-length CeMV sequences showed no apparent aa substitutions suggestive of adaptation to dolphin or whale species. Instead, patterns of non-synonymous substitutions (*dS*) in CeMV-1 characterizing each cluster were identified (Fig. 4). The aa sites 74, 95, and 454 in N; aa site 172 in P; aa site 245 in F; aa sites 121, 256, 422, 451, and 567 in H; and aa sites 122, 136, 399, 873, 1006, 1194, 1196, 1396, and 1698 in L were found to be statistically significant (*p* < 0.05) episodic-positive selection sites using MEME (mixed effects model of evolution)^32^. However, no pervasive positive selection site was identified using an FEL (fixed effects likelihood)^33^. Instead, a greater number of negative selection sites were detected (Supplementary Table S1). Furthermore, the potential variability in SLAM and PVRL-4 binding sites based on MV-H interaction with marmoset SLAM (91% similarity with human SLAM)^34^ and human PVRL-4^35^ were also investigated (Supplementary Table S2). Based on the alignment, cetacean SLAM has approximately 32 and 7% aa differences from human SLAM and PVRL-4, respectively. We identified a number of SLAM interaction sites in the H protein that were conserved among all morbilliviruses, including CeMV, namely D501, D503, D526, S528, R529, P550, Y520, and Y539 (aa position is based on CeMV). For PVRL-4, the sites Y520 (also in SLAM), Y537, Y539, Y454, L460, G461, and D501 (also in SLAM) were found to be conserved.

**Fig. 5:**
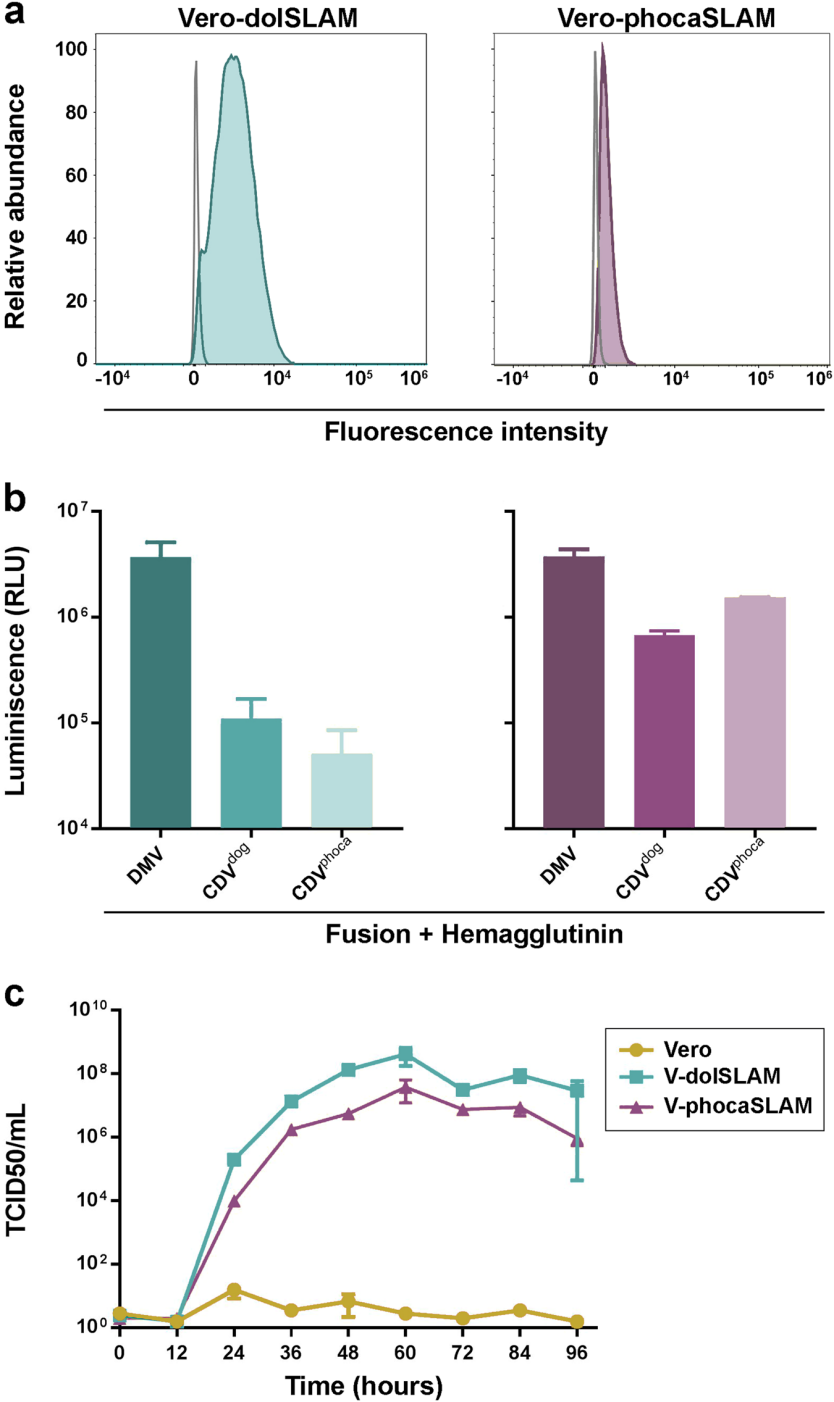
CeMV use of the seal SLAM receptor. **a** Characterization of SLAM expression on Vero cells. Vero-dolSLAM cells (teal colored), Vero-phocaSLAM cells (purple colored) and untransfected Vero cells (gray line) were stained with a HA-tag antibody and analyzed by flow cytometry. **b** Cell-to-cell fusion activity of DMV, CDV-dog/2016, and CDV-phoca/1988 glycoproteins in Vero-dolSLAM cells (left) and Vero-phocaSLAM cells (right) as measured by β-galactosidase activity. Relative luminescence unit (RLU) values on the Y-axis were calculated by subtracting the RLU values obtained from Vero-dolSLAM or Vero-phocaSLAM cells with values generated from Vero cells (control). Experiments were performed in quadruplicates (*n* = 3). **c** Multistep growth curve analyses of DMV-16A inoculated at an moi of 0.001 in Vero cells (control), Vero-dolSLAM and Vero-phocaSLAM cells. Virus titers were determined in triplicates

Previously documented cross-species infections of CeMV in monk and harbor seals^20,22,23^ prompted us to extend our analysis to examine the capacity of CeMV to use phocine SLAM as a cellular receptor. This was evaluated using a quantitative cell-to-cell fusion assay and multistep growth analyses of DMV in Vero cells, newly generated Vero-dolSLAM cells and Vero cells expressing phocine SLAM (Vero-phocaSLAM) (Fig. 5a). DMV F and H glycoproteins induced significantly higher levels of cell-to-cell fusion than the glycoproteins from two CDV strains (of dog and seal origin) in the evaluated cell lines (Fig. 5b), clearly indicating that DMV could use both dolphin and phocine SLAM as cellular receptors. The stringency of the assay was validated by the additional observation that DMV glycoproteins were unable to use human SLAM as a cellular receptor (data not shown). Moreover, when the growth curves were compared (Fig. 5c), DMV growth yielded high titers in both Vero cells expressing SLAM but not in Vero cells. However, DMV showed better replication in Vero-dolSLAM cells compared to Vero-phocaSLAM. Collectively, these results indicate that DMV can readily use the SLAM receptor of seals.

## Discussion

Over the past thirty years, CeMV has emerged as a major pathogen in cetacean species worldwide^3,9,11,13^. During this period, the known host range of CeMV has continually expanded to encompass multiple toothed^3,15,17^ and baleen^5,14,16^ whale species, including endangered populations such as the fin whale^14^. Moreover, instances of spillover into harbor and endangered monk seals have also been documented^20,23^. However, the role of viral molecular adaptation in shaping the evolutionary pathways of CeMV interspecies transmission has remained largely unexplored. In this study, we have used NGS and RACE to determine the first complete CeMV genome sequences from original cetacean tissue samples, including the first complete genome sequence of PMV from a harbor porpoise. We found that CeMV requires minimal genetic variation to maintain multi-host transmission cycles between populations of odontocete and mysticete cetaceans. Further analyses also demonstrated that CeMV can effectively use both dolphin and phocine SLAM as cellular receptors.

Analysis of CeMV sequences enabled us to estimate an overall substitution rate of 2.34 × 10^−4^ subs/site/year, which is lower than what has been proposed for both MV (6.5–8.7 × 10^−4^ subs/site/year)^36^ and CDV (4.8–7.41 × 10^−4^ subs/site/year)^37,38^ based on their H gene sequences. In future, additional full-length sequences of CeMV strains, especially BWMV, PWMV, and GDMV, will help to further calibrate the substitution rate generated in this study. The Bayesian reconstruction of CeMV evolutionary history has indicated that the DMV strain involved in the Mediterranean outbreak in 1990–1992 was more basal to the variants that have emerged within this clade in subsequent years (as also confirmed by maximum likelihood analysis), including the source of the later DMV epizootic in the Mediterranean Sea in 2006–2008. Of particular interest were the DMV variant recovered from a fin whale stranded along the North Sea coast in 2016 and the recently published sequences of CeMV strains from dolphins in the Gulf of Mexico^39^, as these sequences were also derived from a common ancestor. This finding suggests that undefined cetacean species may mediate the trans-oceanic spread of CeMV, driving the dispersal and evolution of this virus, as previously proposed. Pilot whales^40^ and melon-headed whales (*Peponocephala electra*)^41^ have been suggested as viral vectors due to their extensive patterns of migration and their interaction with certain dolphin populations. However, the identity of the hosts acting as vectors that were responsible for triggering CeMV epizootics is a question that requires additional research. Moreover, it remains to be determined if porpoises and/or other cetacean species are the true hosts of PMV, given that this strain has only been previously detected in European waters and the northwest Atlantic Ocean without re-emergence since the last documented case in 1996^22^. In other morbilliviruses such as MV, only 8/24 known genotypes have been detected since 2009^42^, suggesting that many genotypes no longer circulate. Therefore, it is reasonable to speculate that more CeMV strains are likely to be discovered, as shown by the recent detection of the novel strains BWMV and GDMV, and some classical strains, such as PMV, may go unnoticed or disappear.

With respect to viral ecology, anthropogenic factors may have impacted the global ecology and evolution of CeMV. High levels of commercial whaling from the eighteenth to twentieth centuries decimated populations of targeted cetacean species including fin, minke (*Balaenoptera acutorostrata*), humpback (*Megaptera novaeangliae*), and sperm whales. This may well have created bottlenecks for CeMV evolution by reducing cetacean populations to levels that were unable to sustain CeMV endemicity. However, high levels of persistent environmental pollutants, including organochlorine pesticides and polychlorinated biphenyls, especially in the northern Atlantic Ocean, have also been speculated to lead to an increased susceptibility of aquatic mammals to morbillivirus infection^43^.

The divergence of DMV sequences derived from North Sea white-beaked dolphins from the Mediterranean 1990 DMV sequences supports the endemic circulation of this variant in the white-beaked dolphin population, as suggested previously^24^, even before the first documented epizootic in 1990–1992 in the Mediterranean Sea. This implies that the MRCA resided in an unknown cetacean species from the North Atlantic Ocean in the mid-1970s. Furthermore, the numerous aa substitutions identified as unique to this clade might be indicative of virus adaptation to this unknown host species. The population size of white-beaked dolphins in the northeastern Atlantic and North and Barents Seas^44^ might be large enough to sustain endemic transmission, supporting previous findings in which the pathogenicity of this DMV variant was reportedly reduced in this species^24^.

The full-length genomes of DMV and PMV had a divergence of approximately 14%, which is higher than the previously observed variations between CDV strains (5–9%). However, under the current International Committee on Taxonomy of Viruses classification system, DMV and PMV are still considered as CeMV strains, as only CeMV is officially recognized as a species. Moreover, the diversity between recognized morbilliviruses is much higher, ranging between 25 and 54% at the nucleotide level. The genetic evidence presented in this study supports and extends previous observations that the transmission cycle of CeMV, in common with other animal morbilliviruses such as CDV, involves spread between multiple host species. However, this has resulted in a rather arbitrary development of CeMV strain classification. The ability of CeMV strains (e.g., DMV and BWMV) to infect multiple species from both toothed and baleen whale populations creates confusion as the original given strain names are related to the species of first identification. Therefore, we propose to rename the currently known CeMV strains DMV, PMV, PWMV, BWMV, and GDMV to CeMV-1, CeMV-2, CeMV-3, CeMV-4, and CeMV-5, respectively.

In an attempt to investigate the genetic variations driving adaptive evolution of CeMV, we highlighted the molecular footprints characterizing each DMV subgroup. We were unable to identify species-specific variations because no shared mutation was found in a comparison between viruses retrieved from two infected fin whales (DMV-DK/2016 and DMV-Bph). In contrast, the identified aa substitutions were either unique or pertained to their own monophyletic group, regardless of their host species. For instance, full CeMV genome sequences from a fin whale (DMV-Bph) and a striped dolphin (DMV-156), both from the same clade, were highly similar, with 99.9% shared pairwise sequence identity. However, we cannot discard the possibility that DMV-Bph might represent a direct spillover case from an infected dolphin. Additionally, the two fin whale cases differ with respect to age and pathology^14,16^. Further analyses showed evidence that a number of aa sites were under episodic-positive selection, indicating instances of selective pressures that affected only a small subset of DMV sequences within a branch. However, no single pervasive positive selection site was detected in contrast to the large number of sites under purifying selection (negative selection). Purifying selection is a major driver of evolution, removing mutations that would otherwise be deleterious. This process is particularly notable in RNA viruses, which have a high mutation rate due to error-prone virus replication. This selection might explain the low genetic diversity found among DMV variants in this study, further implying that DMV can switch between hosts belonging to both cetacean subgroups (Mysticeti and Odontoceti) and sustain transmission without adaptive evolution. Interestingly, strong purifying selection was also observed in an analysis of rabies virus genomes^45^. This virus predominantly infects members of the order *Carnivora* with spillover into other mammalian species, including humans. The potential host range of CeMV may also not be restricted to only one order, given previous incidences of viral spread to monk and harbor seals^20,22,23^. Therefore, we also assessed the ability of CeMV to use phocine SLAM as a cellular receptor. Surprisingly, CeMV efficiently used phocine SLAM for virus entry and cell-cell fusion, suggesting that in addition to PDV and CDV, CeMV may also pose a risk to seal populations.

Research on many viruses infecting marine mammals, including CeMV, is usually limited by the challenges inherent to studying viral replication and transmission in both the marine environment and appropriate in vitro model systems. To facilitate the study of wild-type CeMV strains, we generated Vero cells that constitutively expressed a dolphin SLAM receptor, thus enabling us to compare authentic wild-type sequences with those of historical and recent virus isolates. We found that the sequence of the DMV-16A isolate cultured in newly generated Vero-dolSLAM cells was comparable to that of the wild-type, except at two sites, S459P in P and I873T in L. However, we also found variable allele frequencies at these two sites in all wild-type DMV sequences. Furthermore, we compared historical laboratory-adapted DMV and PMV strains grown in Vero cells to their wild-type counterpart. A number of substitutions were found, some of which have been previously identified in MV studies. For instance, the substitution g.26A>T has been associated with MV vaccine strains^29^ and has been shown to be important for virus replication^46^. The aa substitutions in the M protein, namely E89K (PMV-2990) and R175G (DMV-muc), have also been shown to be important in MV cell culture adaptation^30,31^. In particular, experiments with the E89K substitution in MV have demonstrated that this mutation can facilitate virus infection in Vero cells and cotton rats^31,47^. Interestingly, some morbillivirus vaccine strains also contain these changes at positions 5 and 12 of the leader sequence, which we identified in this study (Supplementary Fig. S1). The significance of these substitutions with respect to delineating wild-type versus laboratory virus isolates should be investigated further.

In summary, it appears that no or minimal adaptive changes are required for CeMV transmission among cetacean species, reinforcing the threat posed by this virus to endangered cetacean populations. However, the capacity of CeMV to jump into non-cetacean host species besides pinnipeds remains unknown. A more extensive survey of the interaction of CeMV with heterologous SLAM and Nectin-4 receptors may identify additional species that may be susceptible to CeMV infection. Additional full-genome CeMV sequences, and when possible, more extensive serosurveys among cetacean species, will further illuminate the evolutionary history and trajectories of this virus. This approach will necessitate new cross-disciplinary collaborations to acquire samples from regions where gaps in surveillance currently exist such as the Arctic, Indian, southern Atlantic, and western Pacific Oceans, enabling a more comprehensive global overview of this emerging and re-emerging morbillivirus, which has a major impact on the natural ecology and conservation status of dolphin and whale populations.

## Materials and methods

### Tissue samples and viruses

Tissues from CeMV-infected cetaceans analyzed in this study were previously investigated for the presence of morbilliviruses. The samples PMV-2990/brain^7^, DMV-16A/lung^28^, DMV-muc/lung^28^, DMV-DE/2007/brain^48^, DMV-156/lung, DMV-LA/NL/11.2/lung^24^, DMV-Bph/brain^14,49^, and DMV-DK/2016/lung^16^ were collected from animals stranded in European waters (North Sea and Mediterranean Sea). Other samples included PMV-Ulster/88^2^ and PMV-53^7^_passage 4 (P4), from which no original material could be acquired. We also included virus strains DMV-16A_P7, DMV-muc_P5, and PMV-2990_P4, which were previously isolated in Vero cells (green monkey kidney). In addition, DMV-16A was re-isolated from dolphin lung tissue in Vero-dolSLAM cells. Additional tissue samples from a CDV-infected dog from Germany (CDV-dog/2016) and a CDV-infected Baikal seal (*Pusa sibirica*) from Russia (CDV-phoca/1988) were used as the templates for generating additional plasmids expressing morbillivirus glycoproteins.

### Generation of expression plasmids

A plasmid expressing dolphin SLAM (CD150) from a Pacific white-sided dolphin was constructed by subcloning a synthesized open reading frame (ORF) from a dolphin SLAM (Thermo Fischer Scientifics, Waltham, MA, USA) into the mammalian expression vector pCXN2 (Addgene, Cambridge, MA, USA). The design of the synthetic fragment was analogous to previously published constructs^50^ and thus contained the immunoglobulin Igk leader sequence, followed by the influenza virus HA epitope sequence with a linker sequence to the dolphin SLAM sequence (GenBank Accession No. AB428366) in which the signal sequence was removed (aa 1–18). The signal peptide cleavage site was predicted with signal P 4.1 software^51^. A similar design strategy was used for the construction of a plasmid expressing harbor seal SLAM. In brief, harbor seal SLAM was amplified from harbor seal spleen tissue using previously published primers^52^ and was then Sanger-sequenced (Eurofins Genomics, Munich, Germany). Primers containing sequences flanking the adjacent regions (with a linker sequence on the 5′ side and pCXN2 vector sequences on the 3′ side) were designed for the amplification of harbor seal SLAM sequence (GenBank Accession No. MH430950), in which the signal sequence was removed (aa 1–18). A second fragment containing the immunoglobulin Igk leader sequence, influenza virus HA epitope sequence and linker sequence, flanked by the pCXN2 vector sequence on the 5′ side and seal SLAM sequence on the 3′ side, was amplified. All fragments were assembled using the NEBuilder HiFi DNA assembly master mix (NEB) into pCXN2, which had been predigested with *EcoR*I to generate pCXN2-phoca-SLAM. The F and H glycoproteins from DMV-16A, CDV-dog/2016, and CDV-seal/1988 glycoproteins (GenBank Accession Nos. MH430946-49) were cloned into a pCG expression plasmid (kindly provided by Dr. Jürgen Schneider-Schaulies, Institute of Virology and Immunobiology, Würzburg).

### Cell lines

Vero and 293T cells were cultured in advanced MEM and DMEM media respectively, Media were supplemented with 10% FBS, 1% penicillin/streptomycin, and 1% GlutaMax. Vero cells were transfected either with pCXN2-dolSLAM or pCXN2-phoca-SLAM using standard Lipofectamine 3000 protocols (Thermo Fischer Scientific). At 1 day post-transfection, the cells were cultured in growth media supplemented with 1 mg/ml geneticin G418 (Thermo Fischer Scientific) as a selective antibiotic. Vero-dolSLAM cells and Vero-phocaSLAM cells were sorted into single clones using a MoFlo XDP cell sorter (Beckman Coulter) to ensure an equivalent level of SLAM expression within the cell population. Successfully transfected cells were sorted by staining with anti-HA tag antibody [HA.C5] (1:200; ab18181, Abcam) as the primary antibody and chicken anti-mouse IgG (H + L) Alexa Fluor 488 (1:500; A-21200, Thermo Fischer Scientific) as the secondary antibody. Cell clones were expanded and assessed for SLAM expression using an Attune NxT flow cytometer (Thermo Fischer Scientific).

### Virus infection

CeMV growth kinetics were assessed in Vero, Vero-dolSLAM and Vero-phocaSLAM cells. In brief, cells seeded into six-well plates were inoculated with DMV-16A (virus isolated in Vero-dolSLAM) at a multiplicity of infection (MOI) of 0.001 before 1 ml of OptiMEM media was added to the media and incubated at 37 °C. The combined supernatant and cell-associated virus from triplicate wells of each cell line were harvested every 12 h starting from time point 0 until 96 h (4 days). The virus titer was determined by TCID_50_ assay in Vero-dolSLAM cells. The wells were scored as positive when syncytia formation was observed.

### Cell-to-cell fusion assay

Morbillivirus cell-to-cell spread was assessed in Vero cells expressing either dolphin or harbor seal SLAM using a quantitative fusion assay as previously described^53^, with modifications. Plasmids expressing β-galactosidase alpha and omega fragments were kindly provided by Dr. Imke Steffen (RIZ, Hannover). In brief, 293T cells were transfected using calcium phosphate co-precipitation with 3 µg of β-galactosidase omega-fragment expression plasmid, 1.5 µg of pCG-CeMV-F and 1.5 µg of pCG-CeMV-H per well in a 6-well tray. Media were replaced after 8 h. Vero, Vero-dolSLAM, and Vero-phocaSLAM cells were transfected with 200 ng of β-galactosidase alpha-fragment expression plasmid per well in a 96-well tray using Lipofectamine 3000 reagents (Thermo Fischer Scientific). After 48 h, the media in the wells containing 293T cells was removed, and the cells were scraped and overlaid onto β-galactosidase alpha-fragment-transfected cells. The plates were centrifuged for 5 min at 500×*g* and incubated at 37 °C for 6 h. Cell-to-cell fusion was evaluated using a Galacto-Star™ kit (Thermo Fischer Scientific). Enzyme activity was measured using a TECAN Infinite 200 plate reader.

### Sample preparation

Virus-infected tissue samples were prepared following a viral enrichment and random amplification protocol as previously described^54^, with modifications. In brief, 22–221 mg of freshly frozen tissue samples from each animal and 500 µl of the virus isolates were lysed following three freeze/thaw/homogenization cycles to ensure cell disruption. Homogenates were centrifuged and filtered (0.45 μm). RNA was extracted using TRIzol (Thermo Fischer Scientific) and reverse-transcribed with Superscript IV (Thermo Fischer Scientific) using non-ribosomal hexamers flanked by an adaptor (5′gccggagctctgcagatatc3′). Second-strand cDNA synthesis was performed with Klenow fragment DNA polymerase (New England Biolab [NEB], Ipswich, MA, USA). Random amplification of samples was performed using Phusion polymerase (NEB) and the same primer sequence as above. To determine the amount of CeMV present in the samples before performing NGS, DMV and PMV RT-PCRs were performed as previously described^55^.

### Generation of full-length genomes by next-generation sequencing (NGS)

Samples were prepared according to the Nextera XT DNA Library Prep Kit protocol (Illumina, San Diego, CA, USA) using Qubit fluorometric DNA quantitation and a 2100 Bioanalyzer instrument (Agilent, Santa Clara, CA, USA) to assess quality control of the library. Paired-end sequencing of the library was conducted on an Illumina MiSeq system with the MiSeq Reagent Kit v3 (600 cycles; Illumina). Quality trimming and reference assembly were performed using CLC Genomics Workbench 10 software. A full-length DMV sequence (GenBank Accession No. AJ608288) was used as a reference. To confirm the host identity of each sample, reads were also aligned to bottlenose dolphin, bowhead whale, and green monkey sequences, which were retrieved from Ensembl and the bowhead whale genome resource^56^. Quality control of single reads was performed using Trimmomatic V.0.36^57^. Afterwards, high quality reads were mapped to the reference genomes using Bowtie2 V.2.3.4^58^.

### Completion of sequencing gaps and genome termini

Primers (Supplementary Table S2) were designed to cover regions of genomes for which we were unable to recover complete sequences by NGS (DMV_Bp, LA/NL/11.1, DMV-DE/2007, and PMV-53). Gap regions were amplified by PCR using Phusion polymerase and Sanger-sequenced (Eurofins Genomics). In addition, due to the low quality of reads from formalin-fixed paraffin-embedded brain tissue of sample DMV-DK/16, primer sets were designed to amplify the full-length genome (Supplementary Table S3). A RACE protocol was performed to determine the sequence of both 3′ and 5′ termini of CeMV in all positive tissues and virus isolates. Briefly, to determine the 3′ end or leader sequence, RNA was first polyadenylated on the 3′ end by a poly(A) polymerase (NEB) followed by cDNA generation using a poly(T) adaptor as the primer (5′-gactcgagtcgacatcg(T)_17_-3′). A PCR was next performed using the same poly(T) adaptor as forward primer and a reverse primer designed according to consensus sequences at the 3′ end of CeMV genomes obtained in this study (Supplementary Table S4). To determine the 5′ end or trailer sequence, RNA was first converted to cDNA using a primer designed according to consensus sequences at the 5′ end of CeMV genomes obtained in this study (Supplementary Table S4). A poly(A) tail was then added to the cDNA at the 3′ end by a terminal transferase followed by a PCR using a CeMV primer and a poly(T) adaptor as the reverse primer.

### Sequence analyses

Full-length genomes of morbilliviruses (virus-isolate_GenBank accession no.) DMV-GW2010007A_KU720623, DMV-Bph_MH430938, DMV-156_MH430937, DMV-DK/16_MH430939, DMV-16A_MH430934, DMV-muc_MH430935, DMV-DE/2007_MH430940, DMV-NL/11.2_MH430941, PMV-2990_MH430945, PMV-53_MH430943, PMV-Ulster/88_MH430942, DMV_AJ608288 (used as reference in this study), DMV-631IMMS031711_KU720625, DMV-BCF20110815-LA001_KU720624, MV-Zagreb_AF266290, MV-Mvi/Arizona.USA/11.08/2_JN635406.1, RPV-RBOK_Z30697, RPV-Kabete-O_X98291, PPRV-Nigeria/75/1_X74443, PPRV-CH/HNZM/2014_KM089832, CDV-Onderstepoort_AF305419, CDV-SNP/1994/spottel_hyena_1_KU578255, PDV-Wadden_Sea.NLD/1988_KC802221, and partial phosphoprotein sequences of CeMV strains were retrieved from the NCBI database. Alignments were performed with MAFFTv7.

Bayesian analysis was performed to determine the most recent common ancestor (MRCA) and estimate the rate of nucleotide substitution per site, per year using BEAST v2.4.6 software^59^. The sequences were partitioned into coding regions of N, P, M, F, H, and L and non-coding sequences including the leader, trailer and the gene start and gene end of each gene. The test was run for 50 million generations with sampling at every 1000 steps and the following priors: a coalescent-constant population, strict clock model rate, and Hasegawa–Kishino–Yano as the substitution model. Tracer was used to verify quality of the analysis. The initial 10% of trees were discarded as burn-ins using TreeAnnotator, and the best tree was visualized with FigTree. Partial P gene (300 bp) was analyzed using the MASCOT package^60^ and the same priors as described before. Individual complete genes were analyzed based on maximum likelihood in MEGA7.0 with 1000 bootstraps. Nucleotide substitution models were selected as a best-fit model according to Bayesian information criterion. Transient and pervasive selection sites were detected by MEME and FEL available from the Datamonkey web server (www.datamonkey.org).

## Electronic supplementary material

Supplementary Fig. 1

Supplementary Fig. 2

Supplementary Fig. 3

Supplementary Fig. 4

Supplementary Fig. 5

Supplementary Table 1

Supplementary Table 2

Supplementary Table 3

Supplementary Table 4

Supplementary Table 5

## Data Availability

The sequences generated in this study from full-length CeMV genomes and the *Phoca vitulina* SLAM receptor have been deposited under GenBank accession numbers (MH430932-50). Plasmid sequences, cloning primers, and other data are available upon request.
