# Supplementary material for: Evolutionary evidence for multi-host transmission of cetacean morbillivirus
Source: Emerg Microbes Infect. 2018 Dec 5;7:201. doi: 10.1038/s41426-018-0207-x (PMC6279766; doi:10.1038/s41426-018-0207-x)
Supplement: Supplementary file 1 — Supplementary Fig. 1 [file 41426_2018_207_MOESM1_ESM.pdf]

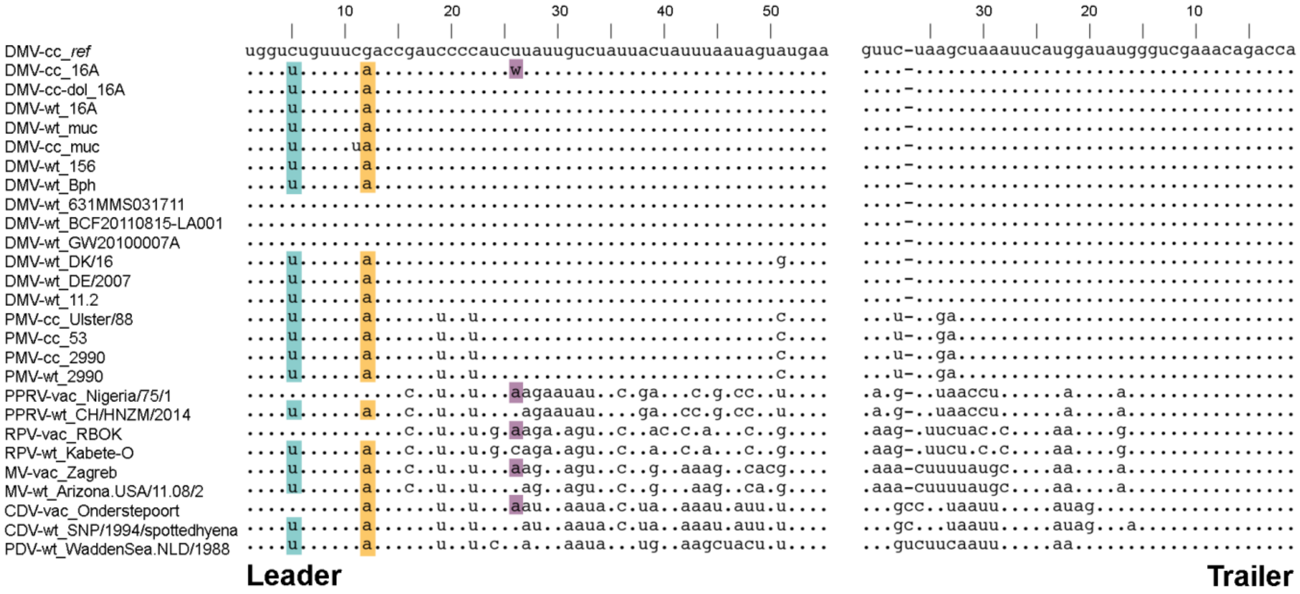

Supplementary Fig. 1 Genome termini alignment of CeMV sequences and other morbilliviruses. Abbreviations: cc, cell-culture; wt, wild-type; vac, vaccine. GenBank accession numbers: DMV-cc_*ref* (AJ608288); DMV-cc_16A (MH430932); DMV-cc_dol_16A (MH430933), DMV-wt_16A (MH430934); DMV-wt_muc (MH430935); DMV-cc_muc (MH430936); DMV-wt_156 (MH430937); DMV-wt_Bph (MH430938); DMV-wt_-631IMMS031711 (KU720625); DMV-wt_BCF20110815-LA001 (KU720624); DMV-wt_GW2010007A (KU720623), DMV-wt_DK/16 (MH430939); DMV-wt_DE/2007 (MH430940); DMV-wt_11.2 (MH430941); PMV-cc_Ulster/88 (MH430942); PMV-cc_53 (MH430943); PMV-cc_2990 (MH430944); PMV-wt_2990 (MH430945); PPRV-vac_Nigeria/75/1 (X74443); PPRV-wt_CH/HNZM/2014 (KM089832); RPV-vac_RBOK (Z30697), RPV-wt_Kabete-O (X98291); MV-vac_Zagreb (AF266290); MV-Mvi/Arizona.USA/11.08/2 (JN635406.1); CDV-vac_-Onderstepoort (AF305419); CDV-wt_SNP/1994/spottel_hyena_1 (KU578255); PDV-wt_Wadden_Sea.NLD/1988 (KC802221).
