# Supplementary material for: Evolutionary evidence for multi-host transmission of cetacean morbillivirus
Source: Emerg Microbes Infect. 2018 Dec 5;7:201. doi: 10.1038/s41426-018-0207-x (PMC6279766; doi:10.1038/s41426-018-0207-x)
Supplement: Supplementary file 2 — Supplementary Fig. 2 [file 41426_2018_207_MOESM2_ESM.pdf]

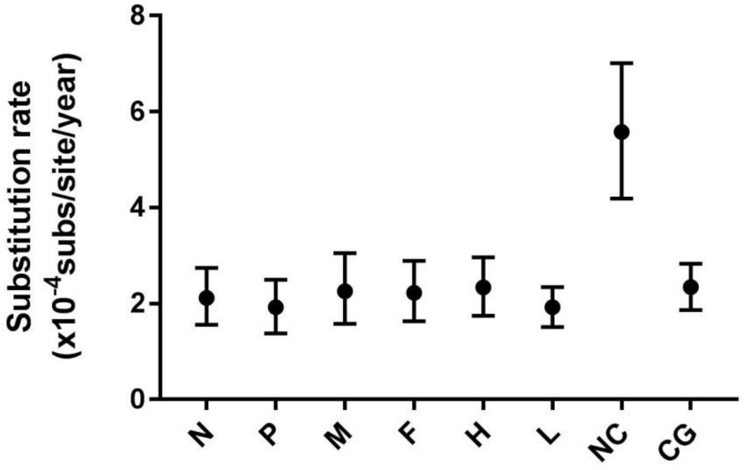

Supplementary Fig. 2 Bayesian estimates of CeMV substitution rates of coding sequences of N, P, M, F, H, and L; non-coding sequences (NC); and complete genome (CG). Values are in x10^{-4} nucleotide substitutions/site/year (highest posterior density interval): N, 2.12 (1.55, 2.74); P, 1.92 (1.38, 2.5); M, 2.56 (1.58, 3.04); F, 2.22 (1.63, 2.89); H, 2.33 (1.74, 2.96); L, 1.93 (1.51, 2.34); NC, 5.58 (4.19, 7.01); CG, 2.34 (1.86, 2.83). Sequences used for estimation (GenBank accession numbers): DMV-Bph (MH430938), DMV-156 (MH430937), DMV-DK/2016 (MH430939), DMV-16A (MH430934), DMV-muc (MH430935), DMV-ref (AJ608288), DMV-DE/2007 (MH430940), DMV-11.2 (MH430941), PMV-2990 (MH430945), PMV-53 (MH430943), PMV-Ulster/88 (MH430942), DMV-Sc/ES/2007 (HQ829973), DMV-Gm/ES/2007 (HQ829972), DMV-631IMMS031711 (KU720625); DMV-BCF20110815-LA001 (KU720624); DMV-GW2010007A (KU720623), and DMV-IZSPLV (MF589987). For L and CG, the sequences AJ608288, HQ829973, HQ829972 were excluded from analyses.
