# Supplementary material for: Evolutionary evidence for multi-host transmission of cetacean morbillivirus
Source: Emerg Microbes Infect. 2018 Dec 5;7:201. doi: 10.1038/s41426-018-0207-x (PMC6279766; doi:10.1038/s41426-018-0207-x)
Supplement: Supplementary file 3 — Supplementary Fig. 3 [file 41426_2018_207_MOESM3_ESM.pdf]

Sequence	34	93	100	107	120	122	127	138	154	180	185	189	318	473	490	508	552
**Sc-ES/1990_AJ608288**	V	D	H	L	I	G	C	S	A	K	T	H	S	R	P	F	Q
Sc-ES/1990|16A	L	D	H	L	I	R	C	G	A	K	T	R	S	R	P	F	K
Sc-ES/1990|muc	L	D	H	L	I	K	C	G	A	K	T	R	S	K	P	F	K
Bp-DK/2016	L	D	H	L	I	K	C	G	A	K	T	R	G	R	P	F	K
Sc-IT/2008|IZSPLV_MF589987	L	D	H	L	I	R	C	G	A	Q	T	R	S	R	P	F	K
Sc-IT/2010|156	L	D	H	L	I	K	C	G	A	K	T	R	S	R	P	F	K
Bp-IT/2013|Bph	L	D	H	L	I	K	C	G	A	K	T	R	S	R	P	F	K
Sl-US/2010|GW2010007A_KU720623	L	D	H	L	I	K	C	G	A	K	T	R	S	R	P	L	K
Tt-US/2011|631MMS031711_KU720625	L	D	H	L	I	K	C	G	A	K	T	R	S	R	L	F	K
Tt-US/2011|BCF20110815-LA001_KU720624	L	D	H	L	I	K	C	G	A	K	T	R	S	R	P	F	K
La-DE/2007	L	A	H	S	I	K	C	G	V	K	I	R	S	R	P	F	K
La-NL/2011|11.2	L	A	Q	L	S	K	G	G	V	K	I	R	S	R	P	F	K

Sequence	639	720	791	811	841	873	1006	1016	1036	1037	1039	1041	1056	1069	1070	1075	1111
**Sc-ES/1990_AJ608288**	G	M	L	L	M	T	V	I	A	D	H	V	R	A	V	L	S
Sc-ES/1990|16A	G	M	L	L	M	I	V	I	D	E	Q	E	I	T	G	I	F
Sc-ES/1990|muc	G	M	L	L	M	T	V	I	D	E	Q	E	I	T	G	I	F
Bp-DK/2016	G	I	L	L	M	T	V	I	D	E	Q	E	I	T	G	I	F
Sc-IT/2008|IZSPLV_MF589987	G	M	L	L	M	T	V	I	D	E	Q	E	I	T	G	I	F
Sc-IT/2010|156	G	M	L	L	M	T	V	I	D	E	Q	E	I	T	G	I	F
Bp-IT/2013|Bph	G	M	L	L	M	T	V	I	D	E	Q	E	I	T	G	I	F
Sl-US/2010|GW2010007A_KU720623	G	M	L	L	T	T	V	I	D	E	Q	E	I	T	G	I	F
Tt-US/2011|631MMS031711_KU720625	G	M	L	L	T	T	V	I	D	E	Q	E	I	T	G	I	F
Tt-US/2011|BCF20110815-LA001_KU720624	G	M	L	L	T	T	V	I	D	E	Q	E	I	T	G	I	F
La-DE/2007	S	M	L	L	M	T	L	V	D	E	Q	E	I	T	G	I	F
La-NL/2011|11.2	S	M	I	V	M	I	L	V	D	E	Q	E	I	K	G	I	F

Sequence	1120	1129	1161	1170	1192	1194	1196	1228	1237	1253	1254	1275	1343	1388	1611	1627	1696
**Sc-ES/1990_AJ608288**	V	R	V	I	G	A	C	A	S	D	Q	R	I	S	Y	S	I
Sc-ES/1990|16A	G	S	D	M	V	S	C	V	A	E	Q	R	I	R	H	S	I
Sc-ES/1990|muc	G	S	D	M	V	S	C	V	A	E	Q	R	I	R	H	S	I
Bp-DK/2016	G	S	D	M	V	S	C	V	A	E	Q	R	I	R	H	S	I
Sc-IT/2008|IZSPLV_MF589987	G	S	D	M	G	A	F	V	A	E	Q	R	I	R	H	S	I
Sc-IT/2010|156	G	S	D	M	V	S	C	V	A	E	Q	R	I	R	H	S	I
Bp-IT/2013|Bph	G	S	D	M	V	S	C	V	A	E	Q	R	I	R	H	S	I
Sl-US/2010|GW2010007A_KU720623	G	S	D	M	V	S	C	V	A	E	Q	R	I	R	H	S	I
Tt-US/2011|631MMS031711_KU720625	G	S	D	M	V	S	C	V	A	E	Q	R	I	R	H	S	I
Tt-US/2011|BCF20110815-LA001 KU720624	G	S	D	M	V	S	C	V	A	E	Q	R	I	R	H	S	I
La-DE/2007	G	S	D	M	V	S	C	V	A	E	R	R	V	R	H	P	T
La-NL/2011|11.2	G	S	D	M	V	S	C	V	A	E	Q	K	V	R	H	P	T

Sequence	1698	1745	1748	1811	1867	1969	1972	1977	2000	2001	2005	2016	2072	2088	2107	2147	2155
**Sc-ES/1990_AJ608288**	A	R	N	R	I	N	G	L	Y	L	I	R	E	A	I	R	P
Sc-ES/1990|16A	A	R	S	S	I	N	G	I	C	I	V	R	E	A	I	K	Q
Sc-ES/1990|muc	A	R	S	S	I	N	G	I	C	I	V	R	E	A	I	K	Q
Bp-DK/2016	A	R	S	S	I	N	G	I	C	I	V	R	E	A	I	K	Q
Sc-IT/2008|IZSPLV_MF589987	A	R	S	S	I	N	G	I	C	I	V	R	E	A	I	K	Q
Sc-IT/2010|156	A	R	S	S	I	N	G	I	C	I	V	R	E	A	I	K	Q
Bp-IT/2013|Bph	A	R	S	S	I	N	G	I	C	I	V	R	E	A	I	K	Q
Sl-US/2010|GW2010007A_KU720623	A	R	S	S	I	N	V	I	C	I	V	I	D	V	I	K	Q
Tt-US/2011|631MMS031711_KU720625	A	R	S	S	I	N	V	I	C	I	V	I	E	V	I	K	Q
Tt-US/2011|BCF20110815-LA001 KU720624	A	R	S	S	I	N	V	I	C	I	V	I	E	V	I	K	Q
La-DE/2007	S	R	S	R	V	N	G	I	C	I	V	R	E	A	N	K	Q
La-NL/2011|11.2	A	K	S	R	V	D	G	I	C	I	V	R	E	A	N	K	Q

Supplementary Fig. 3 Amino acid changes in DMV Large protein. 28/68 changes (green) were accounted for by Sc-ES/1990 (GenBank accession no. AJ608288). Taxon names are presented as host_country/year of collection│variant_GenBank Accession No. Abbreviations: Bp, *Balaenoptera physalus*; Sc, *Stenella coeruleoalba*; Tt, *Tursiops truncatus*; Sl, *Stenella longirostris*; La, *Lagenorhynchus albirostris*; DMV, dolphin morbillivirus; ES, Spain; IT, Italy; US, United States of America; DK, Denmark; DE, Germany; NL, the Netherlands.
