# Supplementary material for: Evolutionary evidence for multi-host transmission of cetacean morbillivirus
Source: Emerg Microbes Infect. 2018 Dec 5;7:201. doi: 10.1038/s41426-018-0207-x (PMC6279766; doi:10.1038/s41426-018-0207-x)
Supplement: Supplementary file 4 — Supplementary Fig. 4 [file 41426_2018_207_MOESM4_ESM.pdf]

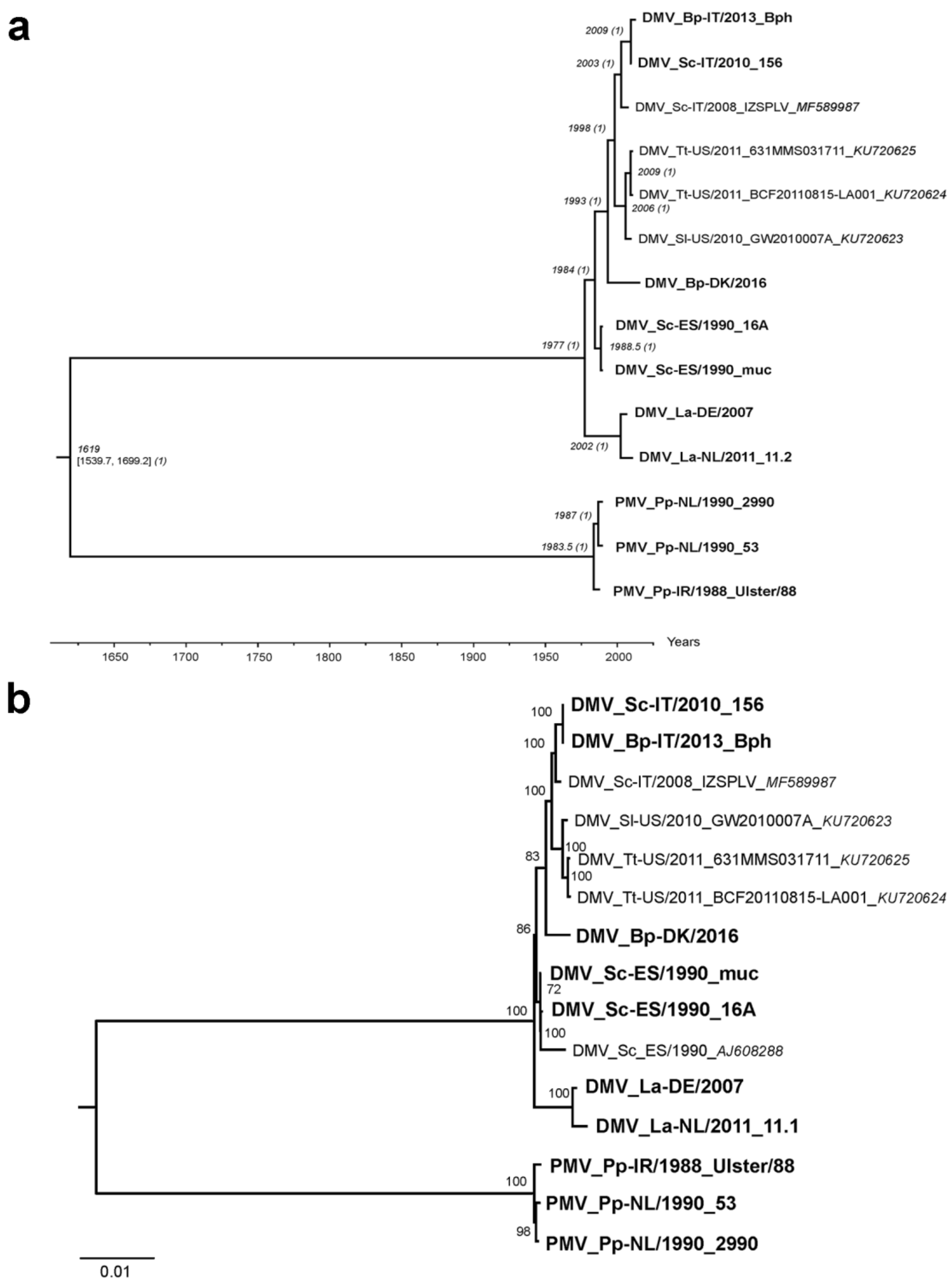

Supplementary Fig. 4 Phylogenetic analyses of full-length CeMVs. **a** Bayesian reconstruction of full-length CeMVs. Ages are presented at nodes, posterior values in parenthesis. **b** Maximum-likelihood reconstruction of full-length CeMVs. Bootstrap values are presented at nodes. Taxon names are presented as virus_host_country/year of collection_variant. GenBank accession number in parentheses: Bph (MH430938), 156 (MH430937), DK/2016 (MH430939), 16A (MH430934), muc (MH430935), DE/2007 (MH430940), 11.2 (MH430941), 2990 (MH430945), 53 (MH430943), Ulster/88 (MH430942). Abbreviations: Bp, *Balaenoptera physalus*; Sc, *Stenella coeruleoalba*; Tt, *Tursiops truncatus*; Sl, *Stenella longirostris*; La, *Lagenorhynchus albirostris*; Pp, *Phocoena phocoena*; DMV, dolphin morbillivirus; PMV, porpoise morbillivirus; ES, Spain; IT, Italy; US, United States of America; DK, Denmark; DE, Germany; NL, the Netherlands; IR, Northern Ireland, U.K.
