# Supplementary material for: Evolutionary evidence for multi-host transmission of cetacean morbillivirus
Source: Emerg Microbes Infect. 2018 Dec 5;7:201. doi: 10.1038/s41426-018-0207-x (PMC6279766; doi:10.1038/s41426-018-0207-x)
Supplement: Supplementary file 5 — Supplementary Fig. 5 [file 41426_2018_207_MOESM5_ESM.pdf]

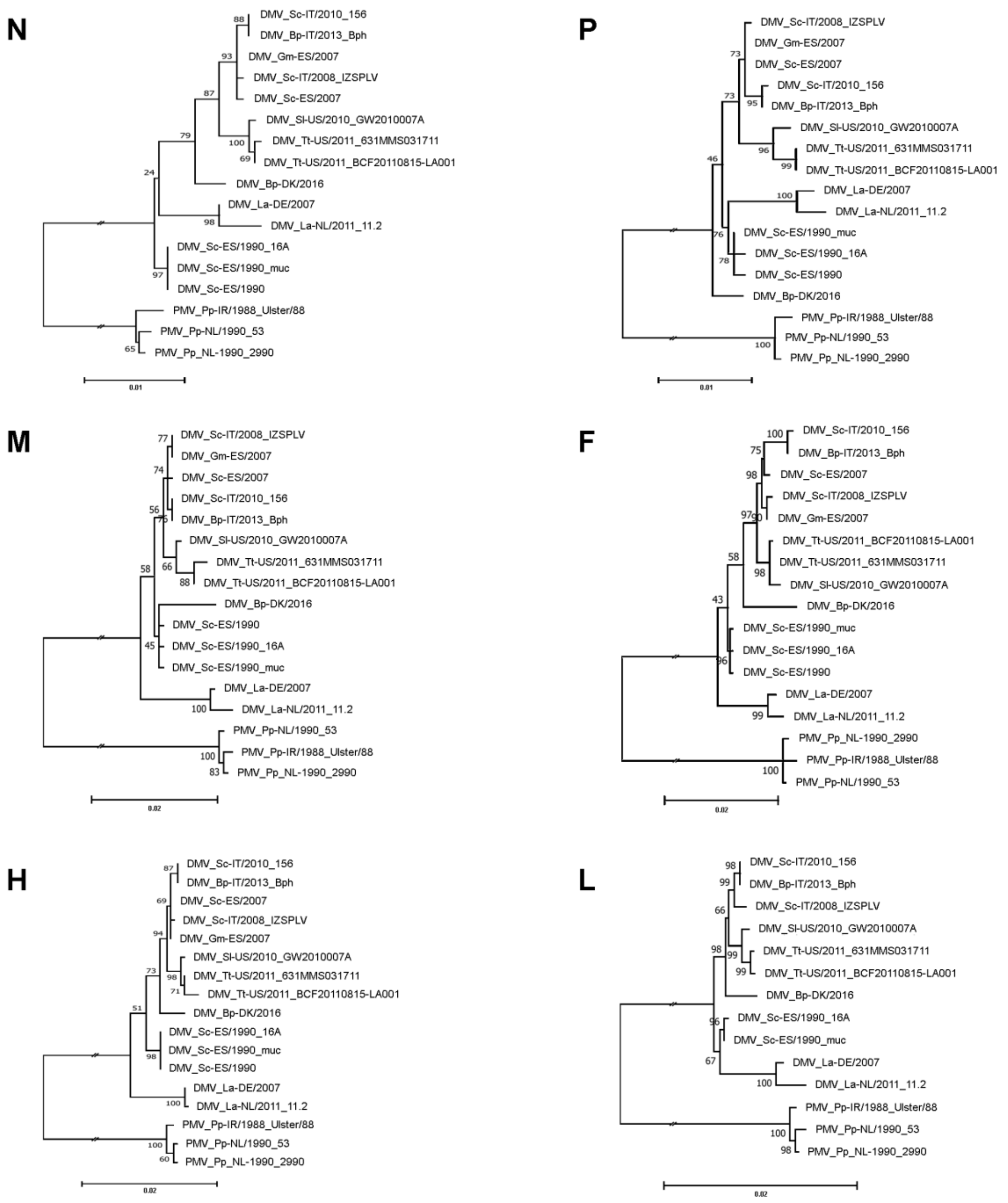

Supplementary Fig. 5 Maximum likelihood reconstruction of individual genes. Bootstrap values at nodes. Taxon names are presented as virus_host_country/year of collection_variant. GenBank accession number in parentheses: Bph (MH430938), 156 (MH430937), IZSPLV (MF589987), 631MMS031711 (KU720625); BCF20110815-LA001 (KU720624), GW2010007A (KU720623), DK/2016 (MH430939), 16A (MH430934), muc (MH430935), DE/2007 (MH430940), 11.2 (MH430941), 2990 (MH430945), 53 (MH430943), Ulster/88 (MH430942). Abbreviations: Bp, *Balaenoptera physalus*; Sc, *Stenella coeruleoalba*; Tt, *Tursiops truncatus*; Sl, *Stenella longirostris*; La, *Lagenorhynchus albirostris*; Pp, *Phocoena phocoena*; DMV, dolphin morbillivirus; PMV, porpoise morbillivirus; ES, Spain; IT, Italy; US, United States of America; DK, Denmark; DE, Germany; NL, the Netherlands; IR, Northern Ireland, U.K.
