# Supplementary material for: Evolutionary evidence for multi-host transmission of cetacean morbillivirus
Source: Emerg Microbes Infect. 2018 Dec 5;7:201. doi: 10.1038/s41426-018-0207-x (PMC6279766; doi:10.1038/s41426-018-0207-x)
Supplement: Supplementary file 6 — Supplementary Table 1 [file 41426_2018_207_MOESM6_ESM.pdf]

Supplementary Table 1 Negative selection sites based on FEL

Protein	Site	Partition	alpha	beta	alpha=beta	LRT	*p*-value	Total branch length
Nucleoprotein	161	1	21,474	0.000	3,089	5,980	0.014	3,116
	103	1	18,114	0.000	3,008	5,675	0.017	2,628
	397	1	11,559	0.000	2,194	5,294	0.021	1,677
	248	1	7,437	0.000	1,728	4,993	0.025	1,079
	470	1	8,020	0.000	1,876	5,046	0.025	1,164
	76	1	17,641	0.000	1,045	4,931	0.026	2,560
	439	1	8,775	0.000	1,912	4,911	0.027	1,273
	319	1	9,182	0.000	1,805	4,840	0.028	1,332
	514	1	44,868	1,483	10,066	4,634	0.031	6,982
	85	1	14,984	0.000	0.819	4,587	0.032	2,174
	34	1	8,120	0.000	1,955	4,514	0.034	1,178
	520	1	16,961	0.000	0.968	4,467	0.035	2,461
	151	1	17,663	0.000	1,032	4,083	0.043	2,563
	38	1	11,171	0.000	0.837	4,023	0.045	1,621
	487	1	20,358	0.000	1,817	4,012	0.045	2,954
	56	1	13,549	0.000	1,031	3,969	0.046	1,966
	63	1	14,905	0.000	1,207	3,969	0.046	2,163
	412	1	6,034	0.000	1,012	3,917	0.048	0.875
	90	1	6,974	0.000	2,397	3,861	0.049	1,012
	283	1	6,034	0.000	1,068	3,877	0.049	0.875
Phosphoprotein	480	1	18,973	0.000	2,621	6,617	0.010	1,618
	103	1	49,500	0.000	1,914	5,441	0.020	4,221
	337	1	20,081	0.000	3,959	5,442	0.020	1,713
	347	1	36,637	0.000	1,937	5,244	0.022	3,124
	190	1	16,486	0.000	5,598	4,844	0.028	1,406
	216	1	14,115	0.000	3,378	4,787	0.029	1,204
	498	1	23,567	0.000	2,954	4,777	0.029	2,010
	396	1	10,971	0.000	3,168	4,416	0.036	0.936
	386	1	11,729	0.000	2,157	4,043	0.044	1,000
	338	1	11,626	0.000	2,254	3,830	0.050	0.992
Matrix	140	1	25156	0.000	5109	7315	0.007	4740
	242	1	17547	0.000	2443	6328	0.012	3306
	169	1	9921	0.000	1952	5210	0.022	1869
	58	1	10901	0.000	1081	4924	0.026	2054
	83	1	8547	0.000	0.986	4979	0.026	1610
	179	1	18199	0.000	0.864	4760	0.029	3429
	279	1	10305	0.000	0.948	4769	0.029	1942
	158	1	7439	0.000	2548	4692	0.030	1402

	259	1	13350	0.000	0.778	4672	0.031	2515
	262	1	13350	0.000	0.795	4630	0.031	2515
	290	1	13561	0.000	0.776	4646	0.031	2555
	253	1	9087	0.000	2129	4575	0.032	1712
	181	1	12855	0.000	0.753	4299	0.038	2422
	280	1	6766	0.000	1838	4321	0.038	1275
	5	1	14152	0.000	0.828	4200	0.040	2667
	211	1	11916	0.000	0.754	4226	0.040	2245
	216	1	10209	0.000	0.604	4160	0.041	1924
	237	1	12472	0.000	0.736	4158	0.041	2350
	302	1	7036	0.000	1066	4168	0.041	1326
	141	1	11841	0.000	0.738	4151	0.042	2231
	117	1	6176	0.000	0.742	4044	0.044	1164
	131	1	8988	0.000	0.594	4012	0.045	1693
	205	1	6176	0.000	0.731	4019	0.045	1164
	272	1	8938	0.000	0.593	4006	0.045	1684
	84	1	5896	0.000	0.736	3974	0.046	1111
	87	1	8988	0.000	0.597	3971	0.046	1693
	294	1	11841	0.000	0.823	3997	0.046	2231
	69	1	6027	0.000	0.922	3897	0.048	1136
	27	1	7986	0.000	0.593	3826	0.050	1505
Fusion	437	1	17483	0.000	1528	8418	0.004	2451
	284	1	18822	0.000	0.883	6146	0.013	2639
	96	1	33271	0.000	1735	5979	0.014	4664
	454	1	36047	0.000	1785	6007	0.014	5054
	160	1	14105	0.000	2608	5897	0.015	1977
	333	1	16036	0.000	2239	5861	0.015	2248
	398	1	15120	0.000	2443	5476	0.019	2120
	138	1	7753	0.000	1136	4948	0.026	1087
	190	1	9270	0.000	1714	4989	0.026	1300
	196	1	9056	0.000	2206	4931	0.026	1270
	448	1	13512	0.000	1263	4906	0.027	1894
	226	1	8679	0.000	1355	4779	0.029	1217
	342	1	7677	0.000	1820	4796	0.029	1076
	400	1	13858	0.000	2070	4651	0.031	1943
	412	1	15893	0.000	1161	4627	0.031	2228
	489	1	7753	0.000	1356	4675	0.031	1087
	517	1	13858	0.000	2070	4651	0.031	1943
	442	1	10453	0.000	2121	4544	0.033	1465
	44	1	13009	0.000	0.691	4266	0.039	1824
	515	1	9853	0.000	3270	4254	0.039	1381

	100	1	12463	0.000	0.994	4159	0.041	1747
	184	1	10387	0.000	1065	4107	0.043	1456
	334	1	9052	0.000	1249	4007	0.045	1269
	514	1	9460	0.000	0.888	4010	0.045	1326
	328	1	15346	0.000	0.907	3971	0.046	2151
	40	1	8880	0.000	0.904	3945	0.047	1245
	394	1	6118	0.000	1921	3898	0.048	0.858
Hemagglutinin	77	1	34493	0.000	0.905	6481	0.011	5606
	97	1	16121	0.000	2608	5812	0.016	2620
	7	1	16730	0.000	2181	5687	0.017	2719
	57	1	11406	0.000	1596	5664	0.017	1854
	575	1	24476	0.000	0.761	5682	0.017	3978
	571	1	12695	0.000	2101	5615	0.018	2063
	282	1	28277	0.000	1011	5429	0.020	4596
	446	1	24476	0.000	0.954	5409	0.020	3978
	43	1	10589	0.000	1214	5227	0.022	1721
	63	1	7578	0.000	1443	5279	0.022	1232
	281	1	21260	0.000	0.953	5275	0.022	3455
	18	1	7531	0.000	1368	5175	0.023	1224
	324	1	23772	0.000	0.717	5135	0.023	3864
	377	1	18423	0.000	0.765	4912	0.027	2994
	529	1	6742	0.000	1647	4811	0.028	1096
	55	1	11263	0.000	2177	4688	0.030	1830
	11	1	18423	0.000	0.899	4654	0.031	2994
	459	1	7578	0.000	1962	4613	0.032	1232
	340	1	7531	0.000	1738	4567	0.033	1224
	349	1	7531	0.000	1738	4567	0.033	1224
	399	1	20008	0.000	0.746	4507	0.034	3252
	508	1	7578	0.000	1785	4519	0.034	1232
	504	1	8034	0.000	1448	4378	0.036	1306
	100	1	12532	0.000	0.697	4127	0.042	2037
	286	1	14890	0.000	1064	4005	0.045	2420
	535	1	21708	0.000	0.837	4020	0.045	3528
Large Protein	1418	1	45.309	0	5.836	11.187	0.001	6.998
	778	1	31.28	0	4.482	9.806	0.002	4.831
	586	1	34.653	0	6.049	9.104	0.003	5.352
	384	1	21.853	0	4.574	8.082	0.004	3.375
	411	1	37.341	0	5.485	8.257	0.004	5.767
	439	1	49.136	0	1.175	7.96	0.005	7.589
	527	1	33.01	0	2.311	7.652	0.006	5.098
	2183	1	42.315	0	1.087	7.697	0.006	6.536

1150	1	19.223	0	3.121	7.325	0.007	2.969
1280	1	80.844	0	0.992	7.236	0.007	12.486
1297	1	18.469	0	4.325	7.312	0.007	2.853
64	1	28.325	0	2.697	6.95	0.008	4.375
99	1	29.628	0	1.455	7.069	0.008	4.576
1772	1	16.775	0	3.557	6.93	0.008	2.591
58	1	15.031	0	3.232	6.823	0.009	2.322
225	1	22.491	0	2.388	6.56	0.01	3.474
1227	1	22.491	0	2.388	6.56	0.01	3.474
1723	1	22.001	0	2.425	6.455	0.011	3.398
2089	1	31.247	0	0.883	6.32	0.012	4.826
310	1	22.351	0	1.606	6.121	0.013	3.452
575	1	17.615	0	2.555	6.179	0.013	2.721
952	1	34.226	0	1.06	6.119	0.013	5.286
954	1	12.479	0	1.52	6.123	0.013	1.927
1811	1	24.175	0	0.728	6.17	0.013	3.734
429	1	17.402	0	2.321	6.051	0.014	2.688
604	1	30.403	0	0.911	6.054	0.014	4.696
1377	1	29.485	0	0.912	6.054	0.014	4.554
2127	1	10.786	0	1.507	5.997	0.014	1.666
2164	1	16.211	0	2.323	5.997	0.014	2.504
420	1	23.208	0	0.983	5.968	0.015	3.585
562	1	29.485	0	1.054	5.912	0.015	4.554
1519	1	24.175	0	0.806	5.972	0.015	3.734
1861	1	24.175	0	0.875	5.894	0.015	3.734
203	1	23.467	0	2.194	5.854	0.016	3.625
1590	1	29.485	0	1.06	5.808	0.016	4.554
293	1	19.522	0	5.83	5.721	0.017	3.015
1632	1	25.676	0	0.726	5.684	0.017	3.966
80	1	10.758	0	1.578	5.578	0.018	1.662
512	1	24.175	0	1.045	5.629	0.018	3.734
1139	1	9.702	0	1.623	5.602	0.018	1.499
406	1	15.714	0	0.938	5.524	0.019	2.427
1158	1	23.601	0	0.805	5.5	0.019	3.645
1193	1	11.096	0	1.531	5.468	0.019	1.714
1483	1	24.175	0	1.052	5.525	0.019	3.734
226	1	22.248	0	0.972	5.436	0.02	3.436
536	1	19.182	0	0.964	5.233	0.022	2.963
1219	1	19.182	0	0.964	5.233	0.022	2.963
1249	1	24.175	0	1.345	5.187	0.023	3.734
1459	1	16.895	0	1.718	5.189	0.023	2.609

1594	1	18.589	0	0.909	5.154	0.023	2.871
394	1	7.726	0	1.014	5.073	0.024	1.193
739	1	11.367	0	2.37	5.069	0.024	1.756
835	1	7.726	0	1.014	5.073	0.024	1.193
1790	1	7.726	0	1.014	5.073	0.024	1.193
2055	1	7.726	0	1.014	5.073	0.024	1.193
2145	1	10.273	0	1.874	5.032	0.025	1.587
405	1	7.369	0	1.02	4.972	0.026	1.138
460	1	12.608	0	3.511	4.923	0.026	1.947
1001	1	7.726	0	1.088	4.931	0.026	1.193
1151	1	7.369	0	1.02	4.972	0.026	1.138
1183	1	6.087	0	1.113	4.938	0.026	0.94
46	1	13.961	0	1.225	4.915	0.027	2.156
934	1	7.726	0	1.103	4.8	0.028	1.193
1259	1	7.369	0	1.078	4.854	0.028	1.138
1648	1	7.369	0	1.078	4.854	0.028	1.138
2037	1	5.779	0	1.1	4.855	0.028	0.893
376	1	23.767	0	1.567	4.689	0.03	3.671
680	1	6.087	0	1.201	4.727	0.03	0.94
714	1	7.369	0	1.095	4.723	0.03	1.138
1097	1	7.369	0	1.095	4.723	0.03	1.138
1550	1	7.369	0	1.095	4.723	0.03	1.138
1082	1	16.584	0	0.995	4.664	0.031	2.561
1213	1	20.627	0	1.002	4.673	0.031	3.186
2050	1	5.792	0	1.188	4.646	0.031	0.895
655	1	7.726	0	1.289	4.611	0.032	1.193
1721	1	6.087	0	1.205	4.596	0.032	0.94
1927	1	22.248	0	1.56	4.611	0.032	3.436
1953	1	7.726	0	1.289	4.611	0.032	1.193
248	1	17.357	0	0.727	4.548	0.033	2.681
1484	1	7.369	0	1.275	4.536	0.033	1.138
359	1	5.792	0	1.193	4.514	0.034	0.895
492	1	5.792	0	1.193	4.514	0.034	0.895
1201	1	11.514	0	1.747	4.505	0.034	1.778
1349	1	11.785	0	0.688	4.499	0.034	1.82
1755	1	11.785	0	0.688	4.499	0.034	1.82
102	1	16.208	0	0.995	4.428	0.035	2.503
205	1	13.794	0	1.028	4.465	0.035	2.131
244	1	17.15	0	0.695	4.463	0.035	2.649
391	1	13.794	0	1.028	4.465	0.035	2.131
1478	1	7.369	0	1.39	4.421	0.035	1.138

1629	1	6.33	0	1.763	4.452	0.035	0.978
2168	1	13.794	0	1.028	4.465	0.035	2.131
1218	1	15.509	0	0.771	4.416	0.036	2.395
587	1	14.836	0	0.693	4.334	0.037	2.291
1476	1	8.292	0	1.327	4.358	0.037	1.281
2156	1	5.594	0	1.708	4.335	0.037	0.864
759	1	17.143	0	0.709	4.325	0.038	2.648
1119	1	13.158	0	0.926	4.321	0.038	2.032
1589	1	20.569	0	0.812	4.306	0.038	3.177
538	1	14.453	0	0.766	4.268	0.039	2.232
626	1	6.469	0	1.971	4.245	0.039	0.999
170	1	17.249	0	0.788	4.2	0.04	2.664
181	1	7.369	0	1.454	4.179	0.041	1.138
351	1	7.369	0	1.454	4.179	0.041	1.138
1072	1	5.988	0	1.127	4.158	0.041	0.925
1173	1	5.988	0	1.127	4.158	0.041	0.925
1178	1	7.369	0	1.454	4.179	0.041	1.138
797	1	15.022	0	0.788	4.116	0.042	2.32
1934	1	17.411	0	0.808	4.125	0.042	2.689
98	1	19.696	0	0.858	4.11	0.043	3.042
511	1	18.341	0	1.027	4.081	0.043	2.833
1375	1	16.735	0	0.807	4.089	0.043	2.585
145	1	17.033	0	0.85	4.057	0.044	2.631
1819	1	18.415	0	0.855	4.04	0.044	2.844
1935	1	13.37	0	0.731	4.001	0.045	2.065
2147	1	32.089	1.177	4.033	3.964	0.046	5.381
1911	1	6.994	0	0.688	3.947	0.047	1.08
1920	1	13.526	0	0.641	3.952	0.047	2.089
118	1	13.37	0	0.802	3.898	0.048	2.065
1399	1	16.722	0	1.018	3.896	0.048	2.583
471	1	6.994	0	0.717	3.868	0.049	1.08
1323	1	6.994	0	0.717	3.868	0.049	1.08
1622	1	6.994	0	0.717	3.868	0.049	1.08
2133	1	13.37	0	0.842	3.83	0.05	2.065
