# Supplementary material for: Evolutionary evidence for multi-host transmission of cetacean morbillivirus
Source: Emerg Microbes Infect. 2018 Dec 5;7:201. doi: 10.1038/s41426-018-0207-x (PMC6279766; doi:10.1038/s41426-018-0207-x)
Supplement: Supplementary file 7 — Supplementary Table 2 [file 41426_2018_207_MOESM7_ESM.pdf]

Supplementary Table 2 Wildtype morbilliviruses Hemagglutinin-SLAM interactions sites

Receptor	H	MV	RPV	PPRV	CeMV		CDV	PDV
					DMV	PMV		
SLAM*	Site I	**D 505**	**D 505**	**D 505**	**D 501**	**D 501**	**D 501**	**D 501**
		**D 507**	**D 507**	**D 507**	**D 503**	**D 503**	**D 503**	**D 503**
	Site II	**D 530**	**D 530**	**D 530**	**D 526**	**D 526**	**D 526**	**D 526**
		T 531	I 531	V 531	T 527	T 527	V 527	V 527
		**S 532**	**S 532**	**S 532**	**S 528**	**S 528**	**S 528**	**S 528**
		**R 533**	**R 533**	**R 533**	**R 529**	**R 529**	**R 529**	**R 529**
		F 552	Y 552	F 552	**F 548**	**L 548**	T 548	T 548
		**P 554**	**P 554**	**P 554**	**P 550**	**P 550**	**P 550**	**P 550**
	Site III	P 191	P 191	R 191	S 191	S 191	A 191	A 191
		T 192	T 192	T 192	V 192	V 192	T 192	T 192
		T 193	T 193	V 193	I 193	I 193	T 193	T 193
		I 194	T 194	T 194	T 194	T 194	S 194	S 194
		R 195	K 195	R 195	R 195	R 195	V 195	M 195
	Site IV	**Y 524**	**Y 524**	**Y 524**	**Y 520**	**Y 520**	**Y 520**	**Y 520**
		Y 541	H 541	Y 541	Y 537	Y 537	Y 537	Y 537
		**Y 543**	**Y 543**	**Y 543**	**Y 539**	**Y 539**	**Y 539**	**Y 539**
		F 552	Y 552	F 552	**F 548**	**L 548**	T 548	T 548
		F 483	L 483	L 483	L 479	L 479	L 479	L 479
		P 545	T 545	T 545	T 541	T 541	P 541	P 541
PVRL4[#]	Site I	**Y 524**	**Y 524**	**Y 524**	**Y 520**	**Y 520**	**Y 520**	**Y 520**
		L 526	S 526	T 526	T 522	T 522	I 522	V 522
		Y 541	H 541	Y 541	Y 537	Y 537	Y 537	Y 537
		**Y 543**	**Y 543**	**Y 543**	**Y 539**	**Y 539**	**Y 539**	**Y 539**
		**P 458**	**P 458**	**P 458**	**P 454**	**P 454**	**P 454**	**P 454**
		M 459	L 459	Y 459	L 455	L 455	K 455	K 455
		L 462	S 462	S 462	S 458	S 458	T 458	T 458
		A 463	A 463	F 463	V 459	V 459	V 459	I 459
		**L 464**	**L 464**	**L 464**	**L 460**	**L 460**	**L 460**	**L 460**
		**G 465**	**G 465**	**G 465**	**G 461**	**G 461**	**G 461**	**G 461**
		L 482	I 482	I 482	T 478	T 478	V 478	I 478
		F 483	L 483	L 483	L 479	L 479	L 479	L 479
	Site II	T 498	T 498	I 498	V 494	V 494	I 494	I 494
		Y 499	Y 499	E 499	E 495	E 495	Q 495	Q 495
		**D 505**	**D 505**	**D 505**	**D 501**	**D 501**	**D 501**	**D 501**
		L 500	L 500	L 500	L 496	L 496	T 496	T 496
	Site III	K 387	R 387	K 387	K 383	K 383	Q 383	K 383
		G 388	D 388	T 388	**S 384**	**G 384**	R 384	R 384
		K 389	R 389	R 389	**R/K 385[§]**	**R 385**	K 385	R 385
		Q 391	P 391	P 391	P 387	P 387	Y 387	Y 387
		Y 499	Y 499	E 499	E 495	E 495	Q 495	Q 495
		L 500	L 500	L 500	L 496	L 496	T 496	T 496

Conserved regions are marked in bold, sites in which DMV and PMV differ are in green
*Sites are based on MV-H interaction with marmoset SLAM (Hashiguchi et al 2011)
[#]Sites are based on MV-H interaction with human nectin-4 (Zhang et al 2013)
[§]In site 385, only variant DMV-DK/16 (North Sea fin whale) has a K instead of R
