# Supplementary material for: Evolutionary evidence for multi-host transmission of cetacean morbillivirus
Source: Emerg Microbes Infect. 2018 Dec 5;7:201. doi: 10.1038/s41426-018-0207-x (PMC6279766; doi:10.1038/s41426-018-0207-x)
Supplement: Supplementary file 8 — Supplementary Table 3 [file 41426_2018_207_MOESM8_ESM.pdf]

Supplementary Table 3 Primers sets covering gap regions of DMV and PMV

Strain	Genome	Gap region (bp)	Forward primer (5' – 3')	Reverse primer (5' – 3')
DMV	DMV/LA/NL/11.2, DE/2007, DMV_Bp	1 – 975	accaRacaaagYtggcta	agaattcatgcaaccctaagg
	DMV_Bp	1290 – 1805	atcagcaggcaaggtgag	agatttgagacactcaagtccc
	DMV/LA/NL/11.2, DE/2007	1929 – 2905	atcaagcgagagtcatcacc	agttctacgtctgcagtaggatc
	DMV/LA/NL/11.2	3856 – 4652	atgctgtcaatcttatcccac	aRcttMtatgtgcgcatgc
	DMV/LA/NL/11.2	5671 – 6257	agataactgcaggagttgcc	ttagatagccattggtcgc
	DMV/LA/NL/11.2	6888 – 7898	aagcctgatctaacaggtactacc	ttgtcatctgcagcacKg
	DMV/LA/NL/11.2, DE/2007	8420 – 9456	agtagcaatcaagactggctg	actgcYtatccctagccttg
	DMV/LA/NL/11.2	9720 – 10201	atggtacttatgtactgcgatgtag	ttcatcatagtctcgtagttgataac
	DMV/LA/NL/11.2	12189 – 12997	atggatagacacatMatcaKacctag	tgttgtatacctcgctactcgg
	DMV/LA/NL/11.2	14479 – 14917	tgtcagtcgagtctagatctgg	aatactagataacactcagtggagat
PMV	53	1459 – 1946	atgccataccaagaggtacaag	tgacgactctcgcttgagtc
