# Supplementary material for: Evolutionary evidence for multi-host transmission of cetacean morbillivirus
Source: Emerg Microbes Infect. 2018 Dec 5;7:201. doi: 10.1038/s41426-018-0207-x (PMC6279766; doi:10.1038/s41426-018-0207-x)
Supplement: Supplementary file 9 — Supplementary Table 4 [file 41426_2018_207_MOESM9_ESM.pdf]

Supplementary Table 4. Primer sets used to amplify and sequence full-length genome of DMV-DK/16

Gap region (bp)	Forward primer (5' – 3')	Reverse primer (5' – 3')	Type of primer
176 – 1325	ttcaggaggagccataagagggattaagcatg	agctctgtcgtcattagcctg	PCR
1220 – 2530	atcagcaggcaaggtgag	cggtctccgtcccagataa	PCR
2442 – 4486	aattccaacctcagtgcc	ggatgaactagctatgagcct	PCR
4046 – 4959	atacatgagcatgatgagagacc	ctctttgcctatgcgagatct	PCR
4939 – 5885	agatctcgcataggcaaagag	aatttctgtcctagcatctcgc	PCR
5791 – 7015	actgtcttagctgttcaaggggt	ctcaggtatgaccaataatccagta	PCR
6888 – 8538	gcctgatctaacaggtactaccaaa	cgactgcagttgtaagggtat	PCR
8331 – 9893	gaggactcaggtctagagcttgat	ggctctaataatgccacgattt	PCR
9767 – 11177	ctgcaatgactgtggacccta	ggacagtgaggatcgctta	PCR
10991 – 12578	aggcttacgagactgtcagtg	cataatgaacaagactcatgacg	PCR
12483 – 14023	cgcaagaggaaggtcgat	tcggttcatcattccaatcc	PCR
13882 – 15702	cttgcagatctgtactgtaatgc	accagacaaagctgggtatag	PCR
662	agatactgcagctgactccgagacg	-	Sequencing
955	ccttagggttgcatgaattctc	-	Sequencing
1750	agtccaaggaattgacctccacatc	-	Sequencing
2174	atgctgacctgctcgtggttcc	-	Sequencing
2953	actgcagacgtagaactcaatcccg	-	Sequencing
3183	gagtgtgcttcgctccatgattaagtc	-	Sequencing
3528	cctcaagttagagttatagatccaggct	-	Sequencing
5400	agtgccagctataaagtgatgaccagg	-	Sequencing
6132	gcatacccgactctatcagaggtcaa	-	Sequencing
6548	agttgaggtagacggaatcacaatcc	-	Sequencing
7379	aagatcatcggagacgaagtgg	-	Sequencing
7802	gacctgatgatccatttgagttccaa	-	Sequencing
8222	actcaagcattgtgcaagagtagaccacc	-	Sequencing
8802	ggcaactctggtgtcatcactactgcat	-	Sequencing
9196	gggtcgagaatggttactccaatca	-	Sequencing
9559	ctgagatgcggtccatcatcaa	-	Sequencing
10179	atcaactacgagactatgatgaagggtc	-	Sequencing
10597	atggctcctatttggaagaccctg	-	Sequencing
11456	aatcctaaggcagcgattgcacg	-	Sequencing
11822	gattaagatggcactactacctgctcc	-	Sequencing
12053	atgtgtccagagtatcactcggc	-	Sequencing
12945	acatcgttaatccgagtagcgagg	-	Sequencing
13248	ccaatcattgagaaggatgcggta	-	Sequencing
13726	atctcacggtctgtaacatgatctaccac	-	Sequencing
14318	gaactcatctgcttgctacaaggca	-	Sequencing
14595	ccagaggtgacatgggtaggtaatgtaga	-	Sequencing
14986	taagaacatcaccgggatttgtagcac	-	Sequencing
15189	gcgtctggatcagagggtcttgtcaa	-	Sequencing
