# Supplementary material for: Evolutionary evidence for multi-host transmission of cetacean morbillivirus
Source: Emerg Microbes Infect. 2018 Dec 5;7:201. doi: 10.1038/s41426-018-0207-x (PMC6279766; doi:10.1038/s41426-018-0207-x)
Supplement: Supplementary file 10 — Supplementary Table 5 [file 41426_2018_207_MOESM10_ESM.pdf]

Supplementary Table 5 RACE primer for DMV and PMV

Strain	Primer	Region (bp)	Primer (5' – 3')
DMV	Leader-outer	291	RV-ggtcaccagcaagtcttactaa
	Leader-inner	142	RV-ttgaacagagctagactccg
	Trailer-outer	15622	FW-tccttgtcagagtgatatcag
	Trailer-inner	15558	FW-aagttgatagggtacggtgc
PMV	Leader-outer	172	RV-attgctcctcctgaacctg
	Leader-inner	103	RV-agaagtgtcgccatgattg
	Trailer-outer	15601	FW-tagaacggatccctactcct
	Trailer-inner	15556	FW-ttaagctgatagggtatggc
-	Adaptor[a]	-	gactcgagtcgacatcg
-	T17 adaptor[a]	-	gactcgagtcgacatcg(T)17

FW = forward; RV = reverse, [a]reference
